# Modulating Lattice Oxygen and Transport Kinetics of Li-Rich Cathodes in All-Solid-State Batteries Through Multifunctional Li_3_ScF_6_ Protective Layer

**DOI:** 10.1007/s40820-026-02209-5

**Published:** 2026-05-21

**Authors:** Peng Lei, Gang Wu, Xiang Qi, Yang Li, Meng Wu, Wanqing Ren, Huan Li, Lei Gao, Dan Zhou, Li-Zhen Fan

**Affiliations:** Institute of Advanced Materials and Technology, University of Science and Technology Beijing, Beijing, 100083 People’s Republic of China

**Keywords:** Li-rich Mn-based oxide cathodes, Li_3_ScF_6_ protective layer, Oxygen redox reversibility, Interfacial transport kinetics, All-solid-state lithium batteries

## Abstract

Li-rich Mn-based oxide (LRMO) cathodes represent promising candidates for high-energy-density all-solid-state lithium batteries (ASSLBs). Nonetheless, irreversible oxygen release and sluggish transport kinetics result in faded voltage and degraded cycling stability, severely impeding their practical applications in ASSLBs. Herein, a high-quality artificial interface layer was constructed on the LRMO surface via a facile sol–gel method followed by thermal treatment, yielding a Li_3_ScF_6_ protective layer comprising a Li_3_ScF_6_ surface coating region and a subsurface Sc doping region. Specifically, Li_3_ScF_6_ surface coating effectively suppresses continuous interfacial side reactions between the cathode and solid electrolyte, thereby improving interfacial transport kinetics; the strong Sc–O bond stabilizes the lattice oxygen framework and inhibits oxygen release, thereby enhancing the reversibility of the oxygen redox reaction. Consequently, the ASSLBs with the modified LRMO cathode exhibit remarkable fast-charging capability (136.8 mAh g^−1^ at 1.0 C) and excellent capacity retention (83.9% after 500 cycles at 0.3 C). In addition, the ASSLBs achieve outstanding long-term cycling stability at a high areal capacity of 4.17 mAh cm^−2^, retaining 81.8% of its capacity after 300 cycles at 60 °C. This study offers new insights into the rational design of high-capacity and high-voltage LRMO cathode materials for high-energy-density ASSLBs.

**Supplementary Information:**

The online version contains supplementary material available at 10.1007/s40820-026-02209-5.

## Highlights

- A multifunctional Li_3_ScF_6_ protective layer is engineered to simultaneously modulate lattice oxygen stability and interfacial transport kinetics of Li-rich cathodes in all-solid-state batteries.
- The Li_3_ScF_6_ layer significantly enhances interfacial contact between Li-rich Mn based oxide and the solid electrolyte, suppressing parasitic interfacial reactions and facilitating faster Li^+^ transport across the interface. Concurrently, the strong Sc–O bonding stabilizes the lattice oxygen framework, suppresses oxygen evolution, and improves the reversibility of oxygen-anion redox.
- The well-configured cells exhibit outstanding electrochemical performance, enabling fast charging, long-term cycling stability, and stable operation at high areal capacities in all-solid-state batteries.

**Supplementary Information:**

The online version contains supplementary material available at 10.1007/s40820-026-02209-5.

## Introduction

Lithium-ion batteries (LIBs) are widely acknowledged as an ideal renewable and clean energy technology capable of displacing fossil fuels and fostering the sustainable development of modern society [1]. Yet, as the demand and deployment of batteries continue to escalate, LIBs employing organic liquid electrolytes encounter formidable challenges in fulfilling the ever-growing requirements for high energy density, enhanced safety, and long-term cycling stability [2, 3]. As a pivotal next-generation electrochemical energy-storage technology, all-solid-state lithium batteries (ASSLBs) have emerged as a particularly promising alternative to conventional LIBs, especially when coupled with high-energy–density cathode materials [4–6]. Among them, Li-rich Mn-based oxide (LRMO) cathodes have attracted considerable attention owing to their unique cationic (transition-metal, TM) and anionic (oxygen) synergistic redox characteristics [7, 8]. In comparison with traditional cathodes, such as LiCoO_2_, LiFePO_4_, and Ni-rich layered oxides, LRMO delivers substantially higher capacities (over 250 mAh g^−1^) and energy densities (~ 1000 Wh kg^−1^) [9, 10], thereby offering broad prospects for application in ASSLBs.

**Fig. 1:**
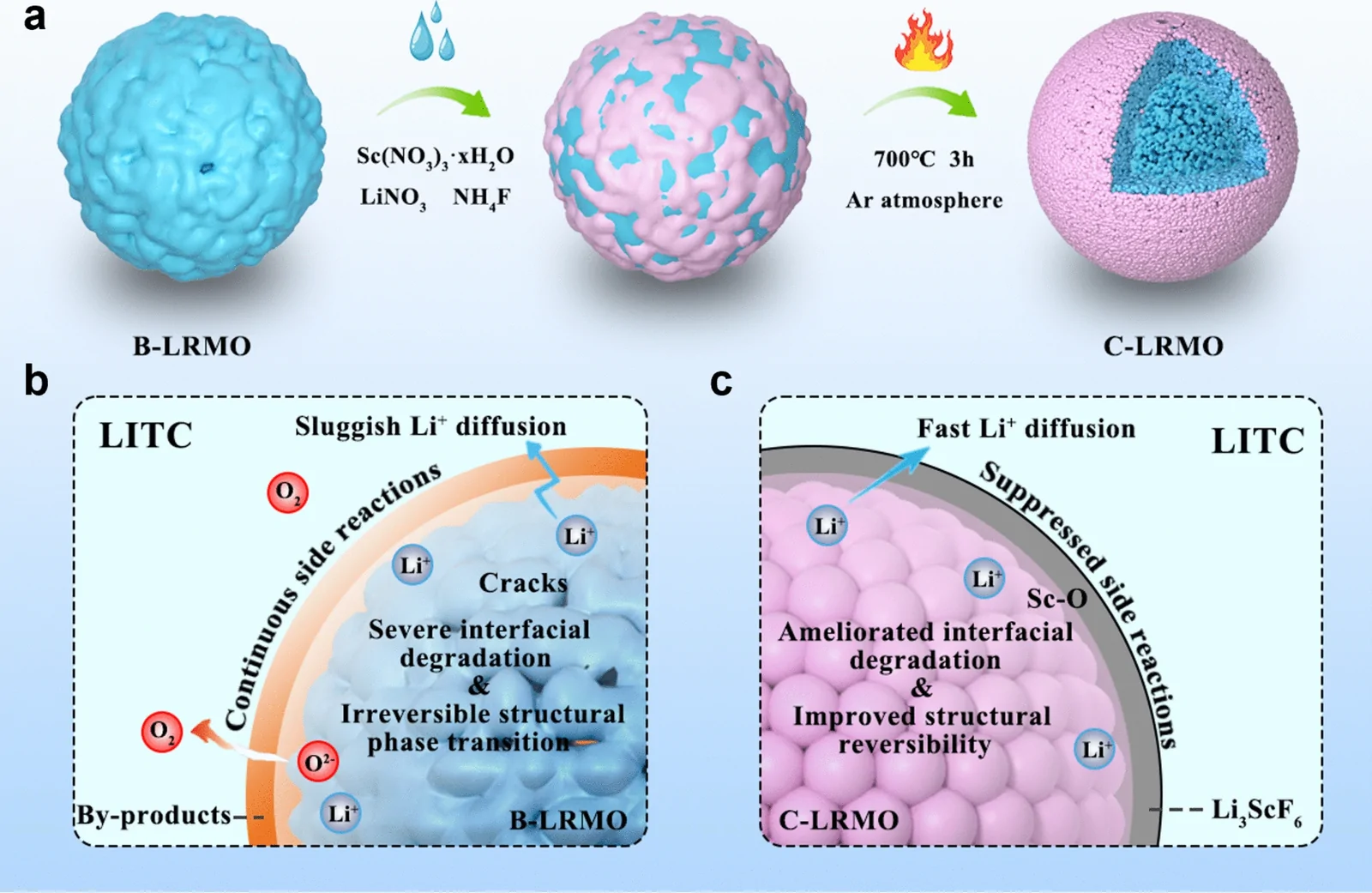
Schematic illustration of **a** the preparation process of C-LRMO, and the operating mechanisms of **b** B-LRMO and** c** C-LRMO in ASSLBs

Although LRMO possesses the advantages of high energy density, elevated voltage plateau, and low Co/Ni contents, its practical application in ASSLBs is hindered by severe kinetic and interfacial challenges, such as sluggish lithium-ion diffusion, irreversible structural phase transformations, and pronounced side reactions between the cathode and the solid electrolyte (SE) (Fig. 1b) [11–13]. Due to the presence of the Li_2_MnO_3_ component in LRMO, its inherent low ionic and electronic conductivity, coupled with high interfacial impedance upon contact with the electrolyte, results in extremely sluggish lithium-ion transport kinetics at the Li_2_MnO_3_/SE interface [14, 15]. In liquid-based batteries, structural degradation of LRMO during cycling mainly originates from bulk volume changes within the material. In contrast, in ASSLBs, the considerably lower wettability of SEs relative to liquid electrolytes impedes intimate interfacial contact between the electrolyte and the electrode [16, 17]. This issue is particularly severe in polycrystalline LRMO composed of aggregated primary particles, where nanopores inaccessible to SEs disrupt ion transport pathways and markedly hinder Li^+^ diffusion [18]. Hence, sustaining a highly interconnected ionic/electronic transport network throughout cycling is essential for activating the anionic redox activity. In addition, interfacial issues also play a critical role in determining the electrochemical performance of LRMO-based ASSLBs. During high-voltage cycling, LRMO is prone to lattice oxygen release, and the reactive O_2_ generated at the surface undergoes severe side reactions with SEs, thereby accelerating the interfacial deterioration with the composite electrode [19, 20]. Concurrently, lattice oxygen release not only undermines the stability of the transition metal–oxygen coordination environment but also drives the gradual transformation of the layered structure into a spinel phase, leading to irreversible structural phase transitions [21, 22]. The accompanying lattice distortions and stress accumulation further compromise the structural integrity of LRMO, ultimately causing rapid capacity fading during prolonged cycling. Therefore, stabilizing the lattice oxygen framework while simultaneously enhancing lithium-ion transport kinetics is essential to improve the electrochemical performance of LRMO in ASSLBs.

Surface coatings (e.g., LiNbO_3_, Al_2_O_3_, Li_3_PO_4_, Li_2_CO_3_, Li_3_BO_3_) are widely recognized as an effective strategy to address the sluggish kinetics and interfacial instability of LRMO [11, 23]. Desirable coating layers should feature excellent structural compatibility with the host cathode while providing high-voltage tolerance and high Li^+^ conductivity. For instance, Sun et al. used atomic layer deposition (ALD) combined with annealing to fabricate an ion-conductive Li_3_PO_4_ coating on LRMO particles. This interphase suppresses lattice oxygen release, retards local structural evolution, mitigates cathode/electrolyte interfacial degradation, and markedly accelerates interfacial lithium-ion transport, thereby sustaining improved charge-transfer kinetics [24]. Similarly, Zhang et al. applied amorphous lithium sulfate treatment to introduce sulfite (SO_3_^2−^) species onto the surface of LRMO, partially replacing oxygen anions. They demonstrated that SO_3_^2−^ participates in charge compensation by accommodating electron transfer from O^2−^ to SO_3_^2−^. This alleviates the peroxidation behavior of surface oxygen during charging, thereby enhancing interfacial stability and enabling ASSLBs to exhibit outstanding stability under long-term cycling [25]. Following a similar rationale, the application of a Li_2_WO_4_ coating onto LRMO not only establishes a stable and rapid ion/electron transport channel at the surface but also significantly lowers the Li^+^ migration barrier and stabilizes the surface oxygen framework. As a result, ASSLBs achieve high areal capacity and outstanding cycling stability in ASSLBs [26]. Despite these advances, most existing coatings are limited to a single barrier function. Ideally, an interfacial modification layer should integrate multiple functionalities: reinforcing cathode structural stability against high-voltage/high-temperature-induced phase transitions and oxygen release; preserving chemical stability at the cathode/electrolyte interface to suppress side reactions; and facilitating rapid Li^+^ transport across the solid-state interface, thereby enabling ASSLBs to achieve both high performance and long cycle life.

Recent computational studies have identified Li_3_ScF_6_ (LSF) as an especially promising protective coating material due to its extremely high anodic limits (~ 6.38 V), high ionic conductivity, and excellent interfacial compatibility with both high-voltage cathodes and halide-based SEs [27]. In this work, an ultrathin LSF protective layer was constructed on the surface of LRMO through a sol–gel approach combined with subsequent annealing (Fig. 1a). The rationally designed interfacial architecture comprises a LSF surface coating layer and a Sc near-surface doping region. Within this composite structure, the LSF coating significantly improves the interfacial contact between LRMO and SE, suppresses side reactions, and substantially promotes lithium-ion transport kinetics. Simultaneously, the strong Sc–O bonds stabilize the lattice oxygen structure, thus mitigating phase transitions induced by high-voltage operation (Fig. 1c). Through a combination of high-resolution transmission electron microscopy (HRTEM), in situ electrochemical impedance spectroscopy (in situ EIS), distribution of relaxation times (DRT) analysis, and density functional theory (DFT) calculations, this work systematically elucidates the significant benefits of the modified LRMO in halide-based ASSLBs, including enhanced lattice oxygen stability, accelerated Li^+^ diffusion kinetics, and substantially improved interfacial stability. As a result, the modified cells deliver outstanding electrochemical performance, featuring an initial discharge capacity of 242.6 mAh g^−1^ at 0.1 C, an excellent rate capability of 136.8 mAh g^−1^ at 1.0 C, and excellent capacity retention of 83.9% after 500 cycles at 0.3 C. Notably, the ASSLBs achieve an ultrahigh areal capacity of 4.17 mAh cm^−2^ at 60 °C and sustain stable operation for 300 cycles at a high cathode loading of 19.1 mg cm^−2^. Notably, although scandium is relatively scarce, the LSF layer in this work is introduced as an ultrathin interfacial coating (~ 1 wt.%), which significantly limits the overall Sc consumption and minimizes its contribution to the total material cost, thereby maintaining practical feasibility for large-scale applications.

## Experimental Section

### Synthesis of Li_2.6_In_0.8_Ta_0.2_Cl_6_ (LITC) and Li_5.3_PS_4.3_ClBr_0.7_ (LPSCB)

For the synthesis of LITC, a stoichiometric mixture of LiCl (Aladdin, 99.99%), InCl_3_ (Aladdin, 99.99%), and TaCl_5_ (Aladdin, 99.99%) was high-energy ball-milled at 600 rpm for 20 h to obtain the precursor, and subsequently annealed at 260 ℃ in a quartz tube for 5 h. For the synthesis of LPSCB, a stoichiometric mixture of Li_2_S (Alfa Aesar, 99.9%), P_2_S_5_ (Macklin, 99%), LiCl (Aladdin, 99.9%), and LiBr (Aladdin, 99.9%) was ball-milled at 500 rpm for 10 h to obtain the precursor, and subsequently annealed at 480 ℃ in a quartz tube for 6 h. All steps above were finished under argon protection to avoid exposure to air.

### Synthesis of C-LRMO

The LSF buffer layers were coated on the bare LRMO particles via a conventional sol–gel method, followed by annealing. For preparing the LSF solution, Sc(NO_3_)_3_·*x*H_2_O (99.9%, Aladdin), LiNO_3_ (99.9%, Aladdin), and NH_4_F (98%, Aladdin) were dissolved in 25 mL of anhydrous ethanol. The pH of the solution was measured to be 3 at 25 °C. Subsequently, 1 g of LRMO powder was slowly added to the prepared solution and subjected to ultrasonication treatment for 30 min. Then, the mixture was magnetically stirred at 60 °C for 2 h and then heated at 80 ℃ for 2 h under vacuum to remove anhydrous ethanol. Based on the mass of LRMO, the mass fraction of LSF in the cathode powder was controlled within the range of 0.5%–2%. Subsequently, the LSF-coated LRMO was synthesized by calcining the LSF-LRMO precursor at 600, 700, and 800 °C for 3 h under an argon atmosphere.

### Materials Characterization

X-ray diffraction (XRD) analysis was carried out using a Rigaku D/max-RB diffractometer with Cu K*α* radiation over a 2*θ* range of 10°–80°. Rietveld refinement was conducted using the GSAS II software. Before testing, all samples were hermetically sealed using polyimide films inside an argon-filled glove box to prevent air exposure. The surface morphology and elemental distribution of the samples were examined using scanning electron microscopy (SEM, HITACHI, SU8100) coupled with energy-dispersive X-ray spectroscopy (EDS). Raman spectra were measured by JY-HR800 using an excitation source at 532 nm. X-ray photoelectron spectroscopy (XPS, ESCALAB 250Xi, Thermo Fisher) with monochromatic Al K*α* radiation as the X-ray source was employed to probe the detailed chemical structure of the samples. All binding energies were calibrated to the C–C peak at 284.8 eV. Spectral deconvolution was performed using Vantage software. X-ray absorption fine structure (XAFS) measurements were carried out at the BL13SSW beamline of the Shanghai Synchrotron Radiation Facility in fluorescence mode, and the data were processed using the Demeter software. Time-of-flight secondary ion mass spectrometry (TOF–SIMS) measurements were conducted over an area of 100 μm × 100 μm using a TOF–SIMS instrument (IONTOF TOF–SIMS M6), with 5 keV Bi^3+^ selected as the primary analysis ion. Negative ion sputtering was performed using Cs^−^ ions at 1 keV with a beam current of 120 nA. High-resolution transmission electron microscopy (HRTEM) images of C-LRMO powders were obtained using a JEOL JEM 2010F field-emission TEM operated at an acceleration voltage of 200  kV, and elemental mapping was conducted via the integrated EDS system.

### Electrochemical Characterizations

The electrochemical impedance spectroscopy (EIS) measurements were carried out using a Solartron electrochemical station (1260 + 1287) under a voltage amplitude of 10 mV, and the frequency range is from 0.1 to 10^6^ Hz. Distribution of relaxation time (DRT) analysis was performed using the MATLAB Graphical User Interface (GUI) toolbox developed by Ciucci's research team (Electrochimica Acta, 2025;184:483-499). To determine the electronic conductivities of the materials, direct current (DC) polarization measurements were conducted by applying a 0.4 V DC bias for 40 min.

### Cell Assembly and Measurements

ASSLBs were assembled using a polyether ether ketone (PEEK) die with a diameter of 10 mm. To prepare the composite cathodes, cathode active materials, LITC, and vapor-grown carbon fiber (VGCF) were ball-milled at 300 rpm for 30 min in a weight ratio of 60:40:5. For cell assembly, 40 mg of LITC powder was compressed into a pellet at 100 MPa. Subsequently, 10 or 25 mg of composite cathode powders were dispersed on one side of the cold-pressed LITC pellet and pressed at 300 MPa. To prevent undesired reactions between LITC and the anode, 60 mg of LPSCB powder was evenly spread on the opposite side of the LITC layer, followed by pressing at 100 MPa. Finally, a piece of Li-In alloy (100 μm) was attached to the LPSCB layer. The battery was kept under a constant pressure of 100 MPa. Prior to electrochemical cycling, the assembled cells were rested for 4 h to allow microstructural equilibration. Galvanostatic charge–discharge measurements were conducted using a LAND battery testing system (CT2001A) with cutoff voltages of 2.2–4.7 V (vs. Li^+^/Li).

### Computational Section

First-principles calculations based on density functional theory (DFT) were carried out through the Vienna Ab initio Simulation Package (VASP) [28]. The generalized gradient approximation (GGA) of the Perdew–Burke–Ernzerhof (PBE) function was applied to describe the exchange–correlation potential [29], and the projector augmented wave (PAW) method [30] was employed to model electron–ion interactions. A plane-wave cut-off energy of 500 eV was set for all calculations. A Monkhorst–Pack *k*-point grid of 3 × 3 × 1 was applied for surface structure calculations. Additionally, the DFT + U approach was employed to better describe the electronic properties and defect states [31]. Total density of states (TDOS) and partial density of states (PDOS) calculations were conducted to analyze the electronic structure of the material. The electron localization function (ELF) was calculated to visualize the spatial distribution of electron density. Bader charge population analysis was used to evaluate charge transfer between atoms. Post-processing of the VASP output data and structural visualizations were carried out using the VASPKIT toolkit [32] and VESTA software[33], respectively.

## Results and Discussion

### Morphology and Structure Characterization

**Fig. 2:**
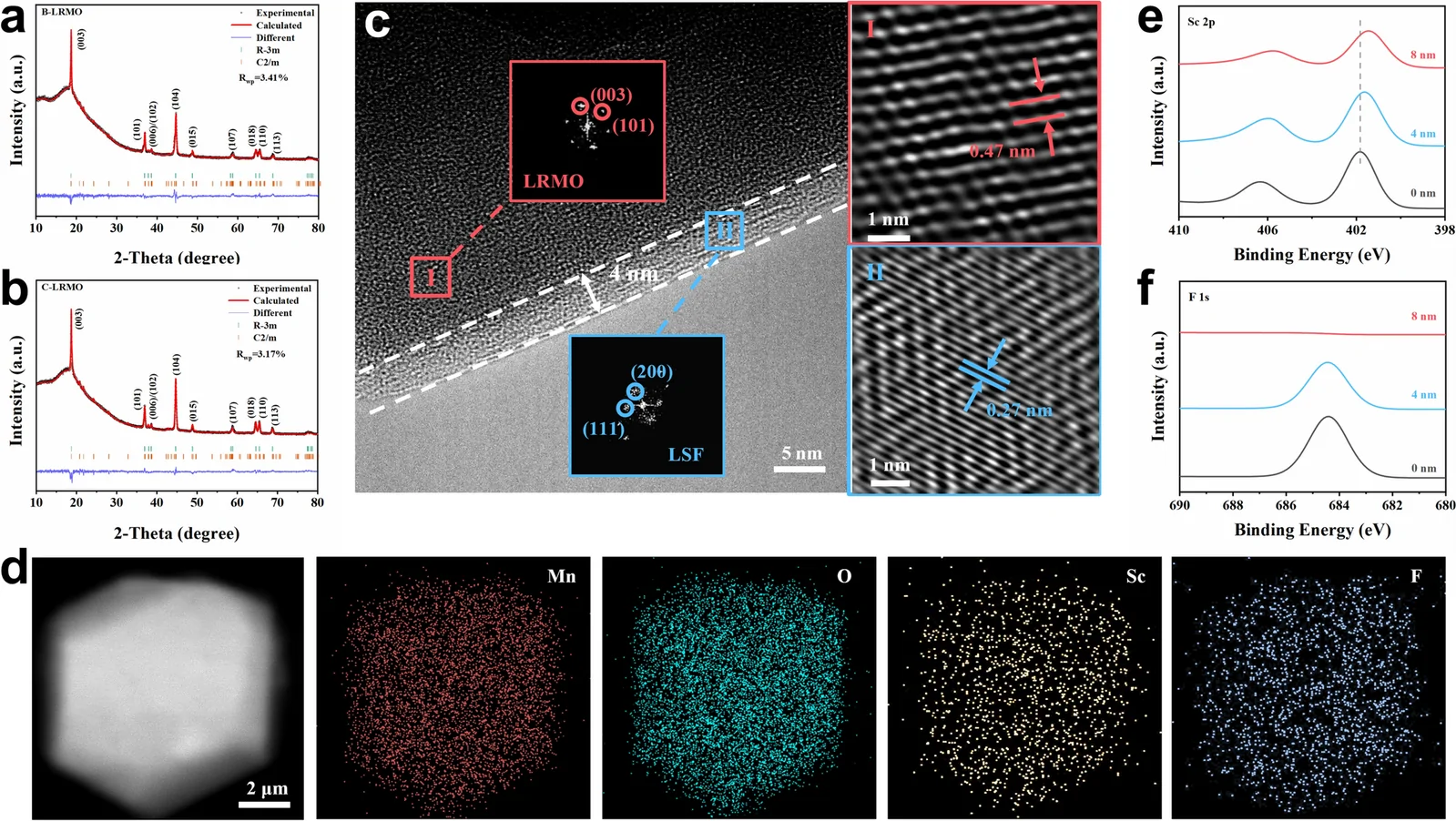
Structure and morphology characterization of B-LRMO and C-LRMO. Rietveld refinement of XRD patterns of **a** B-LRMO and **b** C-LRMO. **c** HRTEM images and corresponding FFT images of C-LRMO. **d** HAADF-STEM images of C-LRMO and corresponding EDS mappings of Mn, O, Sc, and F elements. Depth etching XPS spectrum of **e** Sc 2*p* and **f** F 1*s* in C-LRMO

Li_3_ScF_6_@LRMO powders were synthesized via a facile sol–gel method followed by annealing. The influence of annealing temperature was first investigated. With the Li_3_ScF_6_ coating amount fixed at 1 wt%, we evaluated the electrochemical performance of ASSLBs assembled with LRMO cathodes annealed at 600, 700, and 800 °C. As shown in Fig. S1a, the LRMO annealed at 700 °C exhibits the best rate performance. Therefore, 700 °C was selected as the optimal annealing temperature. Subsequently, with the annealing temperature fixed at 700 °C, the effects of Li_3_ScF_6_ coating levels of 0.5, 1, and 2 wt% were systematically investigated. Among the evaluated compositions, ASSLBs using LRMO cathodes coated with 1 wt% Li_3_ScF_6_ exhibited the best initial discharge capacity, discharge capacity at 0.3 C, and capacity retention at 0.3 C (Fig. S1b, c). Therefore, the LRMO sample coated with 1 wt% Li_3_ScF_6_ and annealed at 700 °C was selected for subsequent studies and denoted as C-LRMO. For comparison, the uncoated LRMO is referred to as B-LRMO. The structural and morphological evolution of the cathode material before and after interfacial modification was investigated using a suite of multi-scale physical characterization techniques. X-ray diffraction (XRD) patterns and corresponding Rietveld refinement results of B-LRMO and C-LRMO (Fig. 2a, b) show that all diffraction peaks can be indexed to a layered *α*-NaFeO_2_-type structure with a R $\overline{3}$ m space group [34, 35]. Additionally, weak superlattice reflections attributable to the C2/m Li_2_MnO_3_ phase are observed in the range of 20°–25°. Notably, after LSF modification, the position of the (003) diffraction peak remains essentially unchanged, accompanied by only slight peak broadening. Meanwhile, the lattice parameters show negligible variation before and after surface modification (Table S1), suggesting that the bulk crystal structure is effectively preserved. Raman spectroscopy (Fig. S2) provides further insights into the structural characteristics. Two prominent peaks located at approximately 596 and 488 cm^−1^ are assigned to the A_1g_ and E_g_ modes of the layered R $\overline{3}$ m structure, respectively. Additionally, several weaker peaks appear in the range of 350–450 cm^−1^, corresponding to the *A*_*g*_ modes of the monoclinic Li_2_MnO_3_ phase [36]. Compared with B-LRMO, C-LRMO exhibits narrower and stronger Raman peaks, further corroborating improved crystallinity.

Scanning electron microscopy (SEM) and transmission electron microscopy (TEM) analyses were conducted on B-LRMO and C-LRMO to obtain detailed morphological and structural features. SEM images (Fig. S3) reveal that the morphology of B-LRMO consists of spherical polycrystalline particles with an average particle size of 10–15 μm. After LSF modification, the particle surfaces become somewhat compact and hazy due to the LSF coating. EDS mapping confirms the uniform distribution of Sc and F elements across the LRMO surfaces, indicating the successful coating of a homogeneous LSF layer on the surface of the LRMO particles (Fig. S4). In order to further investigate the structure of the protective layer on C-LRMO, HRTEM was performed (Figs. 2c and S5). A uniform and dense coating layer with a thickness of ~ 4 nm is observed to conformally cover the surface of LRMO particles. Enlarged images clearly display lattice stripes, which match well with the (111) plane of LSF with a cubic P $\overline{3}$ 1m structure. The interior LRMO exhibits a (003) lattice plane with an interplanar spacing of 0.47 nm, attributable to its layered R $\overline{3}$ m structure [37, 38]. The corresponding fast Fourier transform (FFT) patterns further corroborate the presence of the LSF phase. In addition, HDAAF-STEM EDS mapping reveals a uniform distribution of Ni, Co, Mn, O, Sc, and F elements in C-LRMO, confirming the formation of a homogeneous surface layer (Fig. 2d). The LSF layer establishes an efficient ion-conductive network, which ensures stable and fast interfacial Li^+^ transport kinetics.

The surface chemical states of B-LRMO and C-LRMO were analyzed using X-ray photoelectron spectroscopy (XPS). Distinct signals corresponding to Sc^3+^ and F^−^ are observed in the XPS spectra of C-LRMO (Fig. S6), while the Sc 2*p* and F 1*s* signals are barely detectable in B-LRMO (Fig. S7), confirming the presence of surface LSF coating on C-LRMO. As shown in the O 1*s* XPS spectra (Fig. S8), the binding energy of the O 1*s* peak in C-LRMO shifts to a higher value compared to B-LRMO, accompanied by an increased ratio of lattice oxygen from 56.3% to 67.4%, indicating enhanced stability of the oxygen framework in the cathode [39]. This suggests the near-surface doping of Sc. Then, Ni 2*p*, Co 2*p*, and Mn 2*p* XPS spectra were performed to compare TM valence states of B-LRMO and C-LRMO after LSF coating (Fig. S9a–f). The Ni 2*p* and Co 2*p* spectra reveal negligible changes in their oxidation states, whereas the content of Mn^4+^ increased in C-LRMO. This confirms that Sc atoms are incorporated into the near-surface region, wherein a portion of Mn ions is oxidized to maintain charge balance. The elevated Mn valence is beneficial for suppressing the Jahn–Teller effect and contributes to the improved interfacial structural stability of the C-LRMO cathode [40]. To further demonstrate Sc doping into the subsurface regions of the C-LRMO cathodes, depth etching XPS was performed (Fig. 2e, f). The results show that Sc signals remain detectable up to an etching depth of approximately ~ 8 nm, whereas F signals disappear at similar depths, indicating that F is mainly confined to the outer coating layer while Sc is incorporated into the near-surface lattice. Moreover, the gradual shift of Sc 2*p* binding energy toward lower values with increasing depth suggests the formation of lattice-integrated Sc–O bonding environments. It should be noted that the subsurface Sc distribution represents a gradient region rather than a sharply defined interface. To further verify the formation of Sc–O bonds, the TOF–SIMS depth profiling was performed. As shown in Fig. S10, the ScO^−^ signal is clearly detected, indicating the presence of a Sc–O coordination environment. Notably, the ScO^−^ and MnO^−^ signals exhibit a precise overlap, strongly confirming the uniform distribution of Sc^3+^ within the near-surface region of C-LRMO and the formation of Sc–O bonds. Collectively, these characterizations confirm the successful construction of the strong Sc–O bonds and a stable LSF coating layer in the C-LRMO cathode, which stabilizes lattice oxygen and prevents direct contact between the electrode and SEs, thereby suppressing interfacial side reactions.

### Outstanding Electrochemical Performances of C-LRMO-Based ASSLBs

**Fig. 3:**
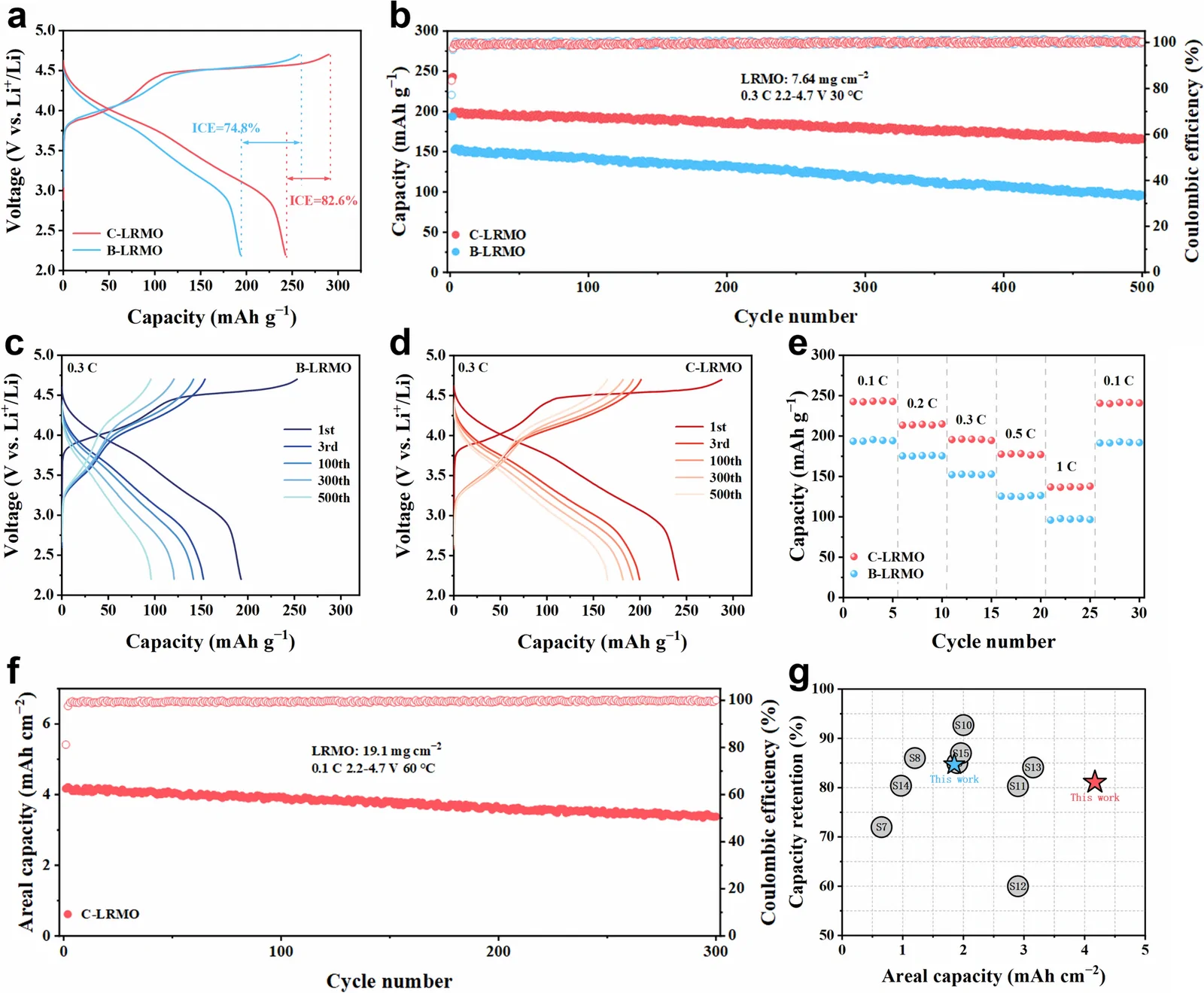
Electrochemical properties of B-LRMO and C-LRMO ASSLBs. **a** First-cycle charge/discharge voltage profiles at 0.1 C. **b** Long-term cycling stability of B-LRMO and C-LRMO at 0.3 C with cathode mass loading of 7.64 mg cm^−2^. Charge–discharge curves of **c** B-LRMO and **d** C-LRMO electrodes at 0.3 C with different cycles. **e** Rate performance of B-LRMO and C-LRMO. **f** Long-term cycling stability at 0.1 C with an ultrahigh cathode mass loading of 19.1 mg cm^−2^. **g** Comparison of areal capacities and capacity retention of C-LRMO with previously reported LRMO materials

To evaluate the impact of surface modification strategy on the applicability of LRMO in ASSLBs, cells were assembled using B-LRMO and C-LRMO cathodes, LITC as the SE, and a LiIn anode. A thin LPSCB layer was placed between the LITC SE layer and the Li–In anode to prevent the reduction of LITC (cell configuration shown in Fig. S11). The ionic conductivity of LITC and LPSCB is 4.2 and 8.9 mS cm^−1^, respectively (Fig. S12). Furthermore, the ionic and electronic conductivities of the composite cathode were assessed through EIS and direct current polarization measurements. As shown in Fig. S13, the C-LRMO composite cathode exhibits markedly elevated ionic conductivity (1.96 × 10^−4^ S cm^−1^) and electronic conductivity (2.92 × 10^−2^ S cm^−1^). Such a highly efficient, bi-continuous electronic/ionic percolation network endows C-LRMO with significantly greater promise for application in ASSLBs. Figure 3a presents the initial charge–discharge profiles of B-LRMO and C-LRMO within the voltage range of 2.2–4.7 V (vs. Li^+^/Li) at 0.1 C (1 C = 250 mAh g^−1^). The C-LRMO exhibits a reversible capacity of 242.6 mAh g^−1^ with an initial Coulombic efficiency (ICE) of 82.6%, whereas the B-LRMO exhibits 193.5 mAh g^−1^ with a lower ICE of 74.8%. Notably, the first charging curve of C-LRMO reveals an extended high-voltage plateau, corresponding to an oxygen redox contribution of 60.9%, which surpasses that of B-LRMO (52.8%) (Fig. S14). The reduced irreversible capacity loss and prolonged oxygen oxidation plateau during the initial charging process indicate enhanced anionic redox reversibility in C-LRMO. This conclusion is further corroborated by cyclic voltammetry (CV), wherein C-LRMO exhibits a significant decrease in polarization from 0.43 to 0.31 V throughout the electrochemical process, which also demonstrates superior redox reversibility for C-LRMO (Fig. S15). Figure 3b–d illustrates the long-term cycling behavior of B-LRMO and C-LRMO-based ASSLBs at 0.3 C. Remarkably, the C-LRMO cell exhibits lower voltage polarization and outstanding capacity retention, sustaining 83.9% of discharge capacity (165.4 mAh g^−1^) after 500 cycles. In contrast, the B-LRMO cell suffers from progressively aggravated voltage polarization and rapid capacity decay fading caused by oxygen release, retaining only 62.4% of its capacity after 500 cycles. Furthermore, as shown in Fig. S16, the critical issue hindering the practical deployment of LRMO cathodes—voltage decay—is markedly mitigated in C-LRMO ASSLBs, with a decay rate of merely 0.32 mV/cycle, in stark contrast to the pronounced 0.56 mV/cycle observed for B-LRMO. This highlights the stable bulk/interfacial structure of C-LRMO. Remarkably, even after 1000 cycles at 1.0 C, the C-LRMO ASSLBs retain 80.4% of their initial capacity (Fig. S17). Kinetic performance serves as a critical indicator of material reliability. Figure 3e compares the rate capabilities of B-LRMO and C-LRMO ASSLBs. C-LRMO delivers reversible discharge capacities of 242.3, 213.5, 195.6, 177.6, and 136.8 mAh g^−1^ at current densities of 0.1, 0.2, 0.3, 0.5, and 1.0 C, respectively, consistently outperforming B-LRMO at the same rates. When the current density is reverted to 0.1 C, the discharge capacity of C-LRMO recovers to 240.8 mAh g^−1^. Correspondingly, the discharge profiles of C-LRMO exhibit markedly lower polarization voltages (Fig. S18). The enhanced rate performance of C-LRMO is attributed to the engineered stable LSF interfacial layer, which effectively suppresses side reactions while facilitating ion transport across the C-LRMO/LITC interface. Notably, C-LRMO-based ASSLBs exhibit excellent electrochemical performance at a high cathode loading of 7.64 mg cm^−2^. To further meet practical requirements, the cathode loading was increased to 19.1 mg cm^−2^ to achieve an areal capacity (~ 3.0 mAh cm^−2^) comparable to industrial standards [41, 42]. Figure 3f exhibits the cell with high areal capacity cycled at 60 °C. The C-LRMO cell delivers an initial discharge capacity of 218.3 mAh g^−1^, corresponding to an areal capacity of 4.17 mAh cm^−2^, and retains 3.38 mAh cm^−2^ after 300 cycles at 0.1 C, achieving a capacity retention of 81.1%. These results underscore its exceptional long-term cycling stability. More importantly, when compared with previously reported works, C-LRMO-based ASSLBs demonstrate the leading level in both areal capacity and capacity retention (Fig. 3g and Table S2), validating the effectiveness of the engineered multifunctional interfacial design.

### Interfacial Transfer Kinetics

**Fig. 4:**
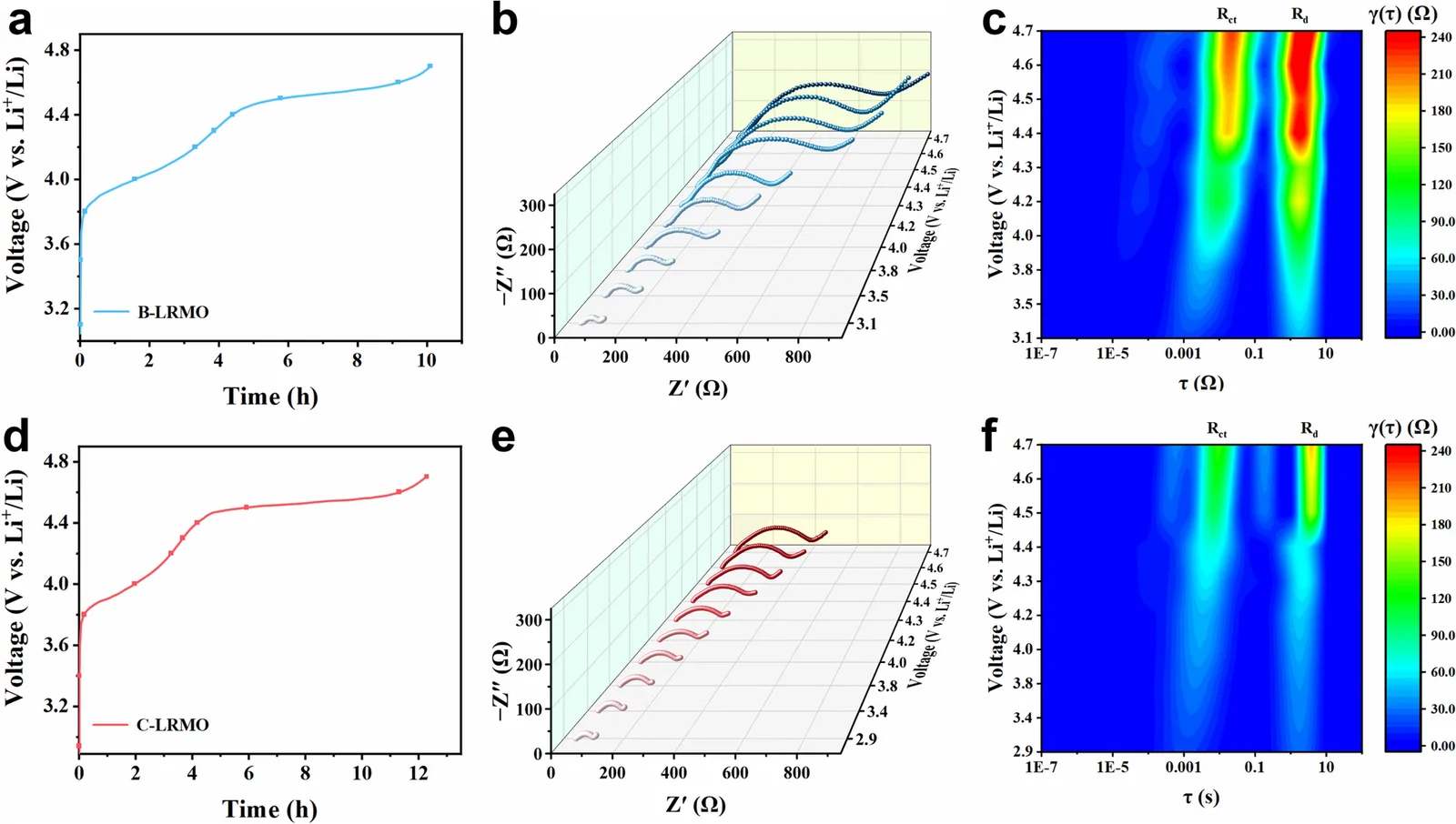
**a** Initial charging voltage profile of the B-LRMO ASSLBs between 2.2 and 4.7 V versus Li^+^/Li. **b** Interfacial impedance evolution of the B-LRMO ASSLBs and **c** corresponding DRT profile transformation derived from in situ EIS. **d** Initial charging voltage profile of the C-LRMO ASSLBs between 2.2 and 4.7 V versus Li^+^/Li. **e** Interfacial impedance evolution of the C-LRMO ASSLBs and **f** corresponding DRT profile transformation derived from in situ EIS

To further elucidate the underlying mechanisms responsible for the enhancement of interfacial kinetics during cycling, the in situ EIS in combination with DRT analysis was employed to evaluate the impedance evolution of B-LRMO and C-LRMO ASSLBs under varying cutoff voltages during the initial charge–discharge cycle (Figs. 4 and S19). It is worth noting that, in comparison to the anode/SE interface, the cathode/SE interfacial resistance dominates the overall cell impedance [43]. Previous studies have demonstrated that the activation of lattice oxygen redox at high voltages can induce the formation of oxidized oxygen species, lattice distortion, and even partial oxygen release from the cathode structure. These processes can significantly deteriorate Li^+^ transport kinetics and promote parasitic interfacial reactions with solid electrolytes, leading to the formation of resistive interphase layers and a substantial increase in interfacial impedance [26, 44–46]. Consistent with these reports, the pronounced impedance increase observed in the B-LRMO ASSLBs above 4.4 V can be attributed to the sluggish interfacial kinetics associated with the activation of anionic redox processes (Fig. 4b). In contrast, C-LRMO cells exhibit a small impedance variation in the mid-to-low frequency region (Fig. 4e), indicating faster and more stable interfacial diffusion kinetics, thereby facilitating more efficient Li-ion transport and mitigating interfacial resistance growth during cycling.

To gain a more comprehensive understanding of the underlying interfacial processes, the DRT analysis was employed to interpret the evolution of EIS spectra and decouple the contributions of distinct electrochemical processes. This method transforms EIS data from the frequency domain into the time domain, yielding a relaxation function* γ*(*τ*) that presents distinct peaks at specific relaxation times [47, 48]. Each peak corresponds to a different electrochemical process, with its area reflecting the associated impedance contribution. Specifically, the peak at 10^−6^ s is attributed to grain boundary resistance of the SEs. The peak between 10^−5^ and 10^−3^ s is associated with lithium-ion transport through the SEI or CEI layer. The peak in the 10^−3^ to 10^−1^ s range corresponds to the charge transfer resistance (*R*_ct_) at the CEI/cathode interface. Finally, the peak observed at 10^0^–10^2^ s is indicative of Li^+^ diffusion resistance (*R*_d_). Figure 4c, f illustrates the conversion of EIS spectra into corresponding DRT functions. It is evident that the *R*_ct_ and *R*_d_ at the B-LRMO/SEs interface increase sharply once the voltage exceeds 4.4 V (Fig. 4c), whereas those at the C-LRMO/SEs interface increase slowly during charging and remain remarkably stable even at 4.7 V (Fig. 4f), suggesting that C-LRMO undergoes less interfacial degradation and exhibits more rapid Li^+^ diffusion kinetics. During the discharge process (Fig. S19), the *R*_ct_ and *R*_d_ of the B-LRMO ASSLBs remain higher than those of the C-LRMO ASSLBs. This further confirms that the LSF coating not only facilitates Li^+^ diffusion but also effectively restrains SEs decomposition induced by oxygen release. Consequently, interfacial Li^+^ transport in ASSLBs is significantly enhanced with LSF coating.

### Interfacial Structural Evolution and Chemical Mechanism

**Fig. 5:**
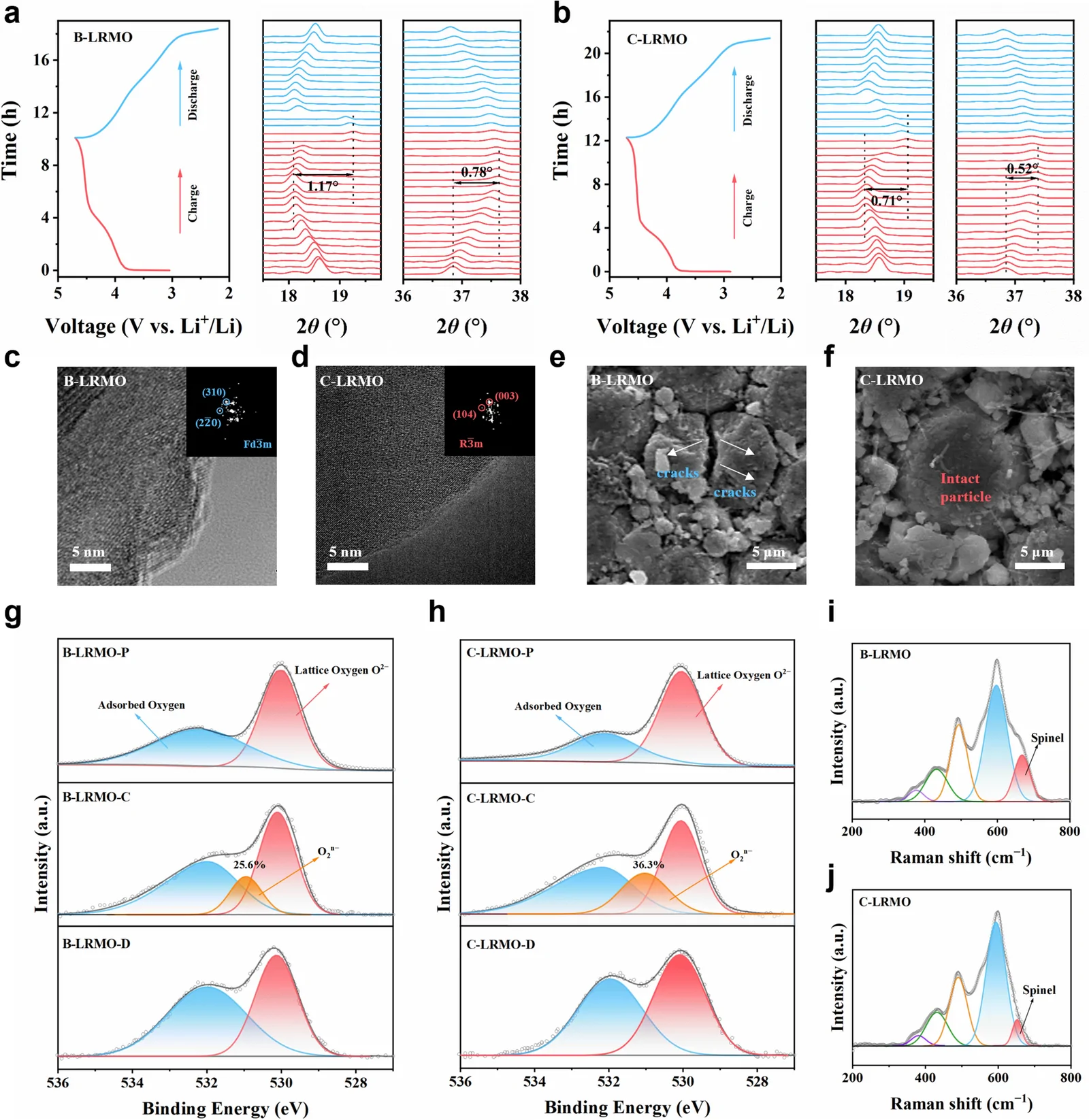
In situ XRD patterns of (003) and (101) diffraction peaks of **a** B-LRMO and **b** C-LRMO during cycling at 2.2–4.7 V. **c, d** TEM and **e, f** SEM images of B-LRMO and C-LRMO after cycling. Ex situ O 1*s* XPS spectra of **g** B-LRMO and **h** C-LRMO at different voltage states. Raman spectra of **i** B-LRMO and **j** C-LRMO after cycling

To further elucidate the underlying mechanism of the LSF coating and its impact on the electrochemical behaviors of cells, in situ XRD measurements were employed to monitor the structural evolution of B-LRMO and C-LRMO during the first charge–discharge cycle, thereby evaluating their structural stability. Figure 5a, b reveals that, prior to the emergence of the voltage plateau, the (003) peak of both samples shifts toward lower angles while the (101) peak moves toward higher angles, indicating lattice expansion along the c-axis and contraction along the a-axis [20]. This evolution corresponds to Li^+^ extraction from the LiNi_1/3_Co_1/3_Mn_1/3_O_2_ phase. After the voltage plateau appears, the (003) peak no longer shifts to lower angles but instead migrates toward higher angles, indicating the activation of the Li_2_MnO_3_ phase and the involvement of oxygen in charge compensation [49]. The associated charge compensation predominantly arises from lattice oxygen oxidation, which results in a significant contraction of the c-axis. During discharge, the (003) peak gradually shifts back toward lower angles as Li^+^ reinserts, accompanied by an increase in peak intensity, indicating the gradual recovery of the layered structure. Compared to B-LRMO, C-LRMO exhibits markedly smaller peak shift during the first cycle, demonstrating that the strong Sc–O bonds formed after LSF coating effectively suppress detrimental lattice distortions and mitigate the accumulation of internal stress, thereby stabilizing the lattice oxygen framework and enhancing the reversibility of the anionic redox process.

SEM and TEM were employed to investigate the structural and morphological evolution of the electrodes after cycling. As shown in Fig. 5c, e, the B-LRMO exhibits severe structural degradation and particle cracking, accompanied by the formation of a substantial amount of spinel phases. This degradation is primarily attributed to detrimental side reactions at the electrode/electrolyte interface. In stark contrast, the C-LRMO retains its complete spherical morphology, with a well-preserved layered bulk structure and a continuous LSF coating (Fig. 5d, f). The presence of the LSF surface layer not only alleviates mechanical stress but also effectively inhibits interfacial reactions between the electrolyte and the oxide cathode during cycling, thereby ensuring structural stability and long-term electrochemical performance under high-voltage conditions. Furthermore, EIS measurements of ASSLBs after 500 cycles (Fig. S20a) reveal a pronounced increase in the impedance values of B-LRMO. DRT analysis (Fig. S20b) indicates that C-LRMO has a smaller *R*_ct_ and *R*_d_ than those of B-LRMO, suggesting that the LSF coating effectively suppresses interfacial degradation, stabilizes the interfacial structure, and enhances interfacial ion transport kinetics under high-voltage conditions.

To elucidate the influence of the LSF coating on the reversibility of anionic redox reactions, the evolution of oxygen species during charge–discharge cycling was systematically investigated. As shown in Fig. 5g, h, compared with the pristine states of B-LRMO and C-LRMO (B-LRMO-P and C-LRMO-P), the O 2*p* XPS spectra of the charged states (B-LRMO-C and C-LRMO-C) exhibit an emergent peak at 530.9 eV, indicating the oxidation of lattice oxygen into O_2_^n−^ species [50]. Notably, the content of the O_2_^n−^ is significantly higher in C-LRMO-C than in B-LRMO-C, suggesting enhanced reversible anionic redox activity in C-LRMO during deep delithiation, with greater retention of O_2_^n−^ species rather than further oxidation to O_2_. In the discharged state (B-LRMO-D and C-LRMO-D), this O_2_^n−^ peak disappears. Moreover, C-LRMO exhibits a markedly higher proportion of the lattice oxygen peak (~ 530.0 eV) relative to B-LRMO, indicating that the LSF coating effectively suppresses lattice oxygen loss. The evolution of In 3*d* and Cl 2*p* signals was further examined to assess interfacial chemical stability. As shown in Fig. S21a, two distinct peaks at 446.1 and 453.6 eV are assigned to In^3+^ species in LITC. For the cycled B-LRMO electrode, the In 3*d* peaks shift toward lower binding energies, approaching those characteristic of In_2_O_3_, suggesting the formation of interfacial byproducts (In_2_O_3_) at the B-LRMO/SEs interface [24]. In contrast, no noticeable binding energy shift is observed for the cycled C-LRMO electrode, highlighting the enhanced interfacial stability imparted by LSF coating. In Fig. S21b, the Cl 2*p* peaks (2*p*_3/2_ at ~ 198.6 eV and 2*p*_1/2_ at ~ 200.2 eV) in cycled B-LRMO undergo significant shape changes and shift to higher binding energies. Conversely, the Cl 2*p* spectral of C-LRMO remains nearly unchanged after cycling, further confirming that the C-LRMO electrode maintains a more stable interface over cycling. Furthermore, X-ray absorption near-edge spectroscopy (XANES) was employed to probe the evolution of the oxygen electronic structure in B-LRMO and C-LRMO during electrochemical cycling (Fig.  S22). Two pre-edge peaks at approximately 529 and 532 eV are observed for both samples, corresponding to the hybridization between O 2*p* and TM 3*d* t_2g_ orbitals and between O 2*p* and TM 3*d* e_g_ orbitals, respectively [51]. In the charged states (B-LRMO-C and C-LRMO-C), the O K-edge XANES spectra display a new peak at 530.8 eV, which is attributed to the characteristic signal of anionic oxygen redox (O_2_^n−^) [52]. Notably, compared with B-LRMO, the O_2_^n−^ signal in C-LRMO becomes more pronounced, indicating enhanced oxygen redox activity and suppressed lattice oxygen loss, which is attributed to the strong Sc–O bonding and the protective effect of the LSF interfacial layer. Upon discharge (B-LRMO-D and C-LRMO-D), the spectral features of C-LRMO almost revert to the pristine state, whereas B-LRMO exhibits incomplete recovery, indicating that the oxygen redox process in C-LRMO is more reversible and that the lattice oxygen framework is more stable [49, 51], which is consistent with the O 1*s* XPS results. Moreover, the Sc K-edge XANES spectra of C-LRMO during electrochemical cycling show that the edge position remains essentially unchanged during electrochemical cycling (Fig. S23a), indicating that Sc does not participate in the electrochemical redox reactions, but instead maintains a stable electronic structure throughout cycling. Furthermore, as shown in Fig. S23b, the Sc K-edge extended X-ray absorption fine structure (EXAFS) spectra exhibit a pronounced peak at approximately 1.5 Å, corresponding to the first coordination shell of Sc–O bonds [53]. The position and intensity of the Sc–O coordination peak remain nearly unchanged throughout electrochemical cycling, indicating the Sc–O bonding network remains highly stable during the cycling process. Such robust Sc–O bonding can effectively reinforce the transition-metal–oxygen framework and suppress irreversible lattice oxygen loss, thereby enhancing the reversibility of the anionic redox reaction and improving the structural stability of the cathode.

We further investigated the structural evolution during electrochemical cycling. Post-cycling ex situ XRD analyses reveal that compared to C-LRMO, the cycled B-LRMO exhibits significantly weakened (003) and (101) peaks, with a shift of 0.25° in the (003) reflection—substantially larger than the 0.13° shift observed for C-LRMO, confirming the superior stability of the oxygen redox process in C-LRMO during cycling (Fig. S24). Furthermore, Fig. 5i, j shows Raman spectra of B-LRMO and C-LRMO after 500 cycles. For B-LRMO, the broad peak located around 660 cm^−1^ is attributed to the distortion of Mn–O polyhedra and contraction of Mn–O bonds, indicating a phase transition from the layered structure to spinel-like structure, triggered by oxygen release during cycling [54]. The fraction of the spinel phase in the cycled C-LRMO electrode (Fig. 5j) is significantly lower than that in the cycled B-LRMO electrode (Fig. 5i), suggesting effective suppression of irreversible phase transition and enhanced oxygen framework stability.

**Fig. 6:**
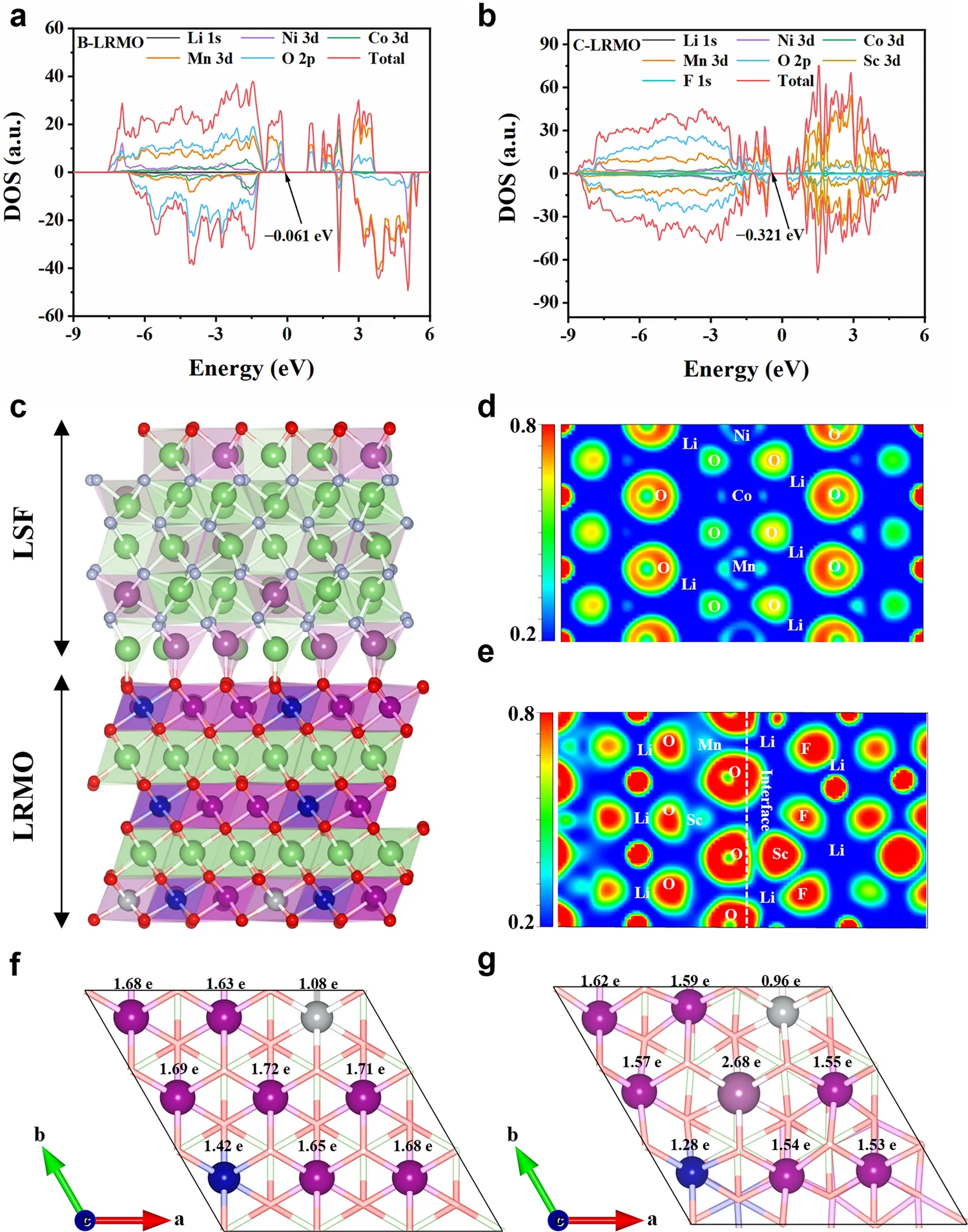
Total density of states (DOS) for **a** B-LRMO and **b** C-LRMO. **c** Relaxed crystal structures of C-LRMO. The color code for atoms: red, O; green, Li; gray, Ni; blue, Co; purple, Mn; orchid purple, Sc; periwinkle blue, F. ELF of valence electrons viewed along the [100] direction for **d** B-LRMO and **e** C-LRMO. Bader charge transfer for **f** B-LRMO and **g** C-LRMO

To comprehensively understand the electronic structure and stability of B-LRMO and C-LRMO, first-principles calculations based on DFT were performed. Figure 6a, b presents the total spin-up and spin-down densities of states (DOS) for transition metals (TMs) and oxygen in both B-LRMO and C-LRMO. The DOS below the Fermi level predominantly arises from filled TM 3*d* and O 2*p* orbitals, corresponding to TM–O bonding and non-bonding O 2*p* states, while the DOS above the Fermi level is mainly attributed to unoccupied TM 3*d* and O 2*p* orbitals. In comparison to B-LRMO, the O 2*p* valence band in C-LRMO exhibits a noticeably higher DOS and shifts 0.321 eV toward lower energy, indicating a reduction in the energy level of the non-bonding O 2*p* states. This electronic modulation substantially suppresses the tendency of lattice oxygen to evolve into O_2_, thereby enhancing both the structural stability of lattice oxygen and the reversibility of anionic redox under high-voltage conditions [55, 56].

The atomic structures of C-LRMO and B-LRMO, depicted in Figs. 6c and S25, respectively, were used for subsequent electronic structure analyses. The electron localization function (ELF) contour maps (Fig. 6d, e) reveal a markedly enhanced degree of electronic localization in C-LRMO, particularly around the TM-O bonds and at the interface. Specifically, the ELF value for the Sc–O bond is higher than that for Mn–O, Ni–O, and Co–O, indicating a stronger Sc–O interaction that contributes to the stabilization of lattice oxygen during lithiation and delithiation. Moreover, the ELF values of O at the C-LRMO interface are significantly higher than those in B-LRMO, suggesting a stronger interfacial electronic constraint that favors enhanced structural stability. The charge density difference map of C-LRMO (Fig. S26) further reveals a pronounced accumulation of electrons on the oxygen atoms at the LSF/LRMO heterointerface, indicating stable lattice O at the interface. Based on the charge density differences, the Bader charge transfer analysis was conducted. As depicted in Fig. 6f, g, the Sc atoms transfer more electrons than Mn, Ni, and Co, which is expected to enhance the stability of surface oxygen. This result is consistent with the previously observed ELF results. First-principles thermodynamic calculations were further performed to evaluate the interfacial reaction energies between both Li_2_MnO_3_ and LiMnO_2_ with LSF and LITC (Fig. S27). The results reveal that the Li_2_MnO_3_/LITC interface exhibits poor thermodynamic stability, with a maximum decomposition energy of −19.6 meV atom^−1^. In contrast, both the Li_2_MnO_3_/LSF, LiMnO_2_/LSF, and LSF/LITC interfaces exhibit negligible reaction energies, indicating high interfacial stability. In addition, thermodynamic equilibrium calculations were performed to further compare the electrochemical windows of LSF and LITC. As shown in Fig. S28, LSF exhibits a high oxidative limit (6.38 V), thereby ensuring excellent interfacial stability with LRMO at high voltages. These findings demonstrate that the LSF protective layer effectively prevents interfacial reactions between LRMO and LITC, thereby ensuring long-term structural integrity of LRMO over prolonged periods.

## Conclusion

In summary, a multifunctional LSF protective layer was successfully constructed on the surface of LRMO cathodes via a simple sol–gel method combined with thermal treatment. This highly oxidation-stable interphase consists of a surface LSF coating layer and a Sc near-surface doping region. The high-quality interphase significantly improves the structural integrity of the cathode and promotes interfacial lithium-ion transport, while effectively mitigating severe interfacial side reactions between the high-voltage cathode and SEs. Therefore, the C-LRMO-based ASSLBs demonstrate exceptional fast-charging capability (136.8 mAh g^−1^ at 1.0 C) and capacity retention (83.9% retention after 500 cycles at 0.3 C). Moreover, the ASSLBs achieve a high areal capacity of 4.17 mAh cm^−2^ at 60 °C and maintain stability over 300 cycles. This study presents a surface modification strategy for LRMO cathodes based on LSF coating with high ionic conductivity and exceptional oxidative stability, offering a promising pathway to develop high-energy-density and high-safety LRMO-based ASSLBs.

## Supplementary Information

Below is the link to the electronic supplementary material.

Supplementary file1 (DOCX 17310 kb)

## References

[1] E.C. Evarts, Lithium batteries: to the limits of lithium. Nature **526**(7575), S93–S95 (2015). 10.1038/526s93a 26509953 10.1038/526S93a

[2] L.-Z. Fan, H. He, C.-W. Nan, Tailoring inorganic–polymer composites for the mass production of solid-state batteries. Nat. Rev. Mater. **6**(11), 1003–1019 (2021). 10.1038/s41578-021-00320-0

[3] P. Lei, G. Wu, H. Liu, X. Qi, M. Wu et al., Boosting ion conduction and moisture stability through Zn^2+^ substitution of chloride electrolytes for all-solid-state lithium batteries. Adv. Energy Mater. **15**(24), 2405760 (2025). 10.1002/aenm.202405760

[4] S. Sun, C.-Z. Zhao, H. Yuan, Y. Lu, J.-K. Hu et al., Multiscale understanding of high-energy cathodes in solid-state batteries: from atomic scale to macroscopic scale. Mater. Futures **1**(1), 012101 (2022). 10.1088/2752-5724/ac427c

[5] A. Manthiram, X. Yu, S. Wang, Lithium battery chemistries enabled by solid-state electrolytes. Nat. Rev. Mater. **2**(4), 16103 (2017). 10.1038/natrevmats.2016.103

[6] M. Weiss, F.J. Simon, M.R. Busche, T. Nakamura, D. Schröder et al., From liquid- to solid-state batteries: ion transfer kinetics of heteroionic interfaces. Electrochem. Energy Rev. **3**(2), 221–238 (2020). 10.1007/s41918-020-00062-7

[7] W. He, W. Guo, H. Wu, L. Lin, Q. Liu et al., Challenges and recent advances in high capacity Li-rich cathode materials for high energy density lithium-ion batteries. Adv. Mater. **33**(50), e2005937 (2021). 10.1002/adma.202005937 33772921 10.1002/adma.202005937

[8] S. Zhao, K. Yan, J. Zhang, B. Sun, G. Wang, Reaction mechanisms of layered lithium-rich cathode materials for high-energy lithium-ion batteries. Angew. Chem. Int. Ed. **60**(5), 2208–2220 (2021). 10.1002/anie.202000262 10.1002/anie.202000262 32067325

[9] P.M. Csernica, K. McColl, G.M. Busse, K. Lim, D.F. Rivera et al., Substantial oxygen loss and chemical expansion in lithium-rich layered oxides at moderate delithiation. Nat. Mater. **24**(1), 92–100 (2025). 10.1038/s41563-024-02032-6 39420105 10.1038/s41563-024-02032-6

[10] M. Zhang, L. Qiu, W. Hua, Y. Song, Y. Deng et al., Formulating local environment of oxygen mitigates voltage hysteresis in Li-rich materials. Adv. Mater. **36**(16), 2311814 (2024). 10.1002/adma.202311814 10.1002/adma.202311814 38194156

[11] W.-J. Kong, C.-Z. Zhao, S. Sun, L. Shen, X.-Y. Huang et al., From liquid to solid-state batteries: Li-rich Mn-based layered oxides as emerging cathodes with high energy density. Adv. Mater. **36**(14), 2310738 (2024). 10.1002/adma.202310738 10.1002/adma.202310738 38054396

[12] S.-L. Cui, M.-Y. Gao, G.-R. Li, X.-P. Gao, Insights into Li-rich Mn-based cathode materials with high capacity: from dimension to lattice to atom. Adv. Energy Mater. **12**(4), 2003885 (2022). 10.1002/aenm.202003885

[13] J. Huang, B. Ouyang, Y. Zhang, L. Yin, D.-H. Kwon et al., Inhibiting collective cation migration in Li-rich cathode materials as a strategy to mitigate voltage hysteresis. Nat. Mater. **22**(3), 353–361 (2023). 10.1038/s41563-022-01467-z 36702887 10.1038/s41563-022-01467-z

[14] K. Hikima, K. Shimizu, H. Kiuchi, Y. Hinuma, K. Suzuki et al., Reaction mechanism of Li_2_MnO_3_ electrodes in an all-solid-state thin-film battery analyzed by operando hard X-ray photoelectron spectroscopy. J. Am. Chem. Soc. **144**(1), 236–247 (2022). 10.1021/jacs.1c09087 34957828 10.1021/jacs.1c09087

[15] N. Hu, Y.-H. Zhang, Y. Yang, H. Wu, Y. Liu et al., Unraveling the spatial asynchronous activation mechanism of oxygen redox-involved cathode for high-voltage solid-state batteries. Adv. Energy Mater. **14**(13), 2303797 (2024). 10.1002/aenm.202303797

[16] L. Xu, S. Tang, Y. Cheng, K. Wang, J. Liang et al., Interfaces in solid-state lithium batteries. Joule **2**(10), 1991–2015 (2018). 10.1016/j.joule.2018.07.009

[17] M. Yan, W.-P. Wang, Y.-X. Yin, L.-J. Wan, Y.-G. Guo, Interfacial design for lithium–sulfur batteries: from liquid to solid. EnergyChem **1**(1), 100002 (2019). 10.1016/j.enchem.2019.100002

[18] S. Payandeh, D. Goonetilleke, M. Bianchini, J. Janek, T. Brezesinski, Single versus poly-crystalline layered oxide cathode materials for solid-state battery applications - a short review article. Curr. Opin. Electrochem. **31**, 100877 (2022). 10.1016/j.coelec.2021.100877

[19] X. Xu, S. Chu, S. Xu, S. Guo, H. Zhou, Self-constructing a lattice-oxygen-stabilized interface in Li-rich cathodes to enable high-energy all-solid-state batteries. Energy Environ. Sci. **17**(9), 3052–3059 (2024). 10.1039/d4ee00938j

[20] D. Luo, H. Zhu, Y. Xia, Z. Yin, Y. Qin et al., A Li-rich layered oxide cathode with negligible voltage decay. Nat. Energy **8**(10), 1078–1087 (2023). 10.1038/s41560-023-01289-6

[21] Y.-J. Guo, P.-F. Wang, Y.-B. Niu, X.-D. Zhang, Q. Li et al., Boron-doped sodium layered oxide for reversible oxygen redox reaction in Na-ion battery cathodes. Nat. Commun. **12**(1), 5267 (2021). 10.1038/s41467-021-25610-7 34489437 10.1038/s41467-021-25610-7 PMC8421359

[22] M. Zhang, L. Qiu, Y. Sun, Y. Song, Z. Wu et al., Microstructure-controlled Li-rich Mn-based cathodes by a gas–solid interface reaction for tackling the continuous activation of Li_2_MnO_3_. ACS Appl. Mater. Interfaces **13**(34), 40995–41003 (2021). 10.1021/acsami.1c12221 34415716 10.1021/acsami.1c12221

[23] J. Liang, Y. Zhu, X. Li, J. Luo, S. Deng et al., A gradient oxy-thiophosphate-coated Ni-rich layered oxide cathode for stable all-solid-state Li-ion batteries. Nat. Commun. **14**(1), 146 (2023). 10.1038/s41467-022-35667-7 36627277 10.1038/s41467-022-35667-7 PMC9832028

[24] R. Yu, C. Wang, H. Duan, M. Jiang, A. Zhang et al., Manipulating charge-transfer kinetics of lithium-rich layered oxide cathodes in halide all-solid-state batteries. Adv. Mater. **35**(5), e2207234 (2023). 10.1002/adma.202207234 36461688 10.1002/adma.202207234

[25] S. Sun, C.-Z. Zhao, H. Yuan, Z.-H. Fu, X. Chen et al., Eliminating interfacial O-involving degradation in Li-rich Mn-based cathodes for all-solid-state lithium batteries. Sci. Adv. **8**(47), eadd5189 (2022). 10.1126/sciadv.add5189 36427308 10.1126/sciadv.add5189 PMC9699669

[26] W.-J. Kong, C.-Z. Zhao, L. Shen, S. Sun, X.-Y. Huang et al., Bulk/interfacial structure design of Li-rich Mn-based cathodes for all-solid-state lithium batteries. J. Am. Chem. Soc. **146**(41), 28190–28200 (2024). 10.1021/jacs.4c08115 39354739 10.1021/jacs.4c08115 PMC11488500

[27] J. Liu, S. Wang, Y. Qie, Q. Sun, Identifying lithium fluorides for promising solid-state electrolyte and coating material of high-voltage cathode. Mater. Today Energy **21**, 100719 (2021). 10.1016/j.mtener.2021.100719

[28] G. Kresse, J. Furthmüller, Efficient iterative schemes for *ab initio* total-energy calculations using a plane-wave basis set. Phys. Rev. B **54**(16), 11169–11186 (1996). 10.1103/physrevb.54.11169 10.1103/physrevb.54.11169 9984901

[29] J.P. Perdew, K. Burke, M. Ernzerhof, Generalized gradient approximation made simple. Phys. Rev. Lett. **77**(18), 3865–3868 (1996). 10.1103/physrevlett.77.3865 10062328 10.1103/PhysRevLett.77.3865

[30] P.E. Blöchl, Projector augmented-wave method. Phys. Rev. B **50**(24), 17953–17979 (1994). 10.1103/physrevb.50.17953 10.1103/physrevb.50.17953 9976227

[31] M. Bajdich, M. García-Mota, A. Vojvodic, J.K. Nørskov, A.T. Bell, Theoretical investigation of the activity of cobalt oxides for the electrochemical oxidation of water. J. Am. Chem. Soc. **135**(36), 13521–13530 (2013). 10.1021/ja405997s 23944254 10.1021/ja405997s

[32] V. Wang, N. Xu, J.-C. Liu, G. Tang, W.-T. Geng, VASPKIT: a user-friendly interface facilitating high-throughput computing and analysis using VASP code. Comput. Phys. Commun. **267**, 108033 (2021). 10.1016/j.cpc.2021.108033

[33] K. Momma, F. Izumi, VESTA 3 for three-dimensional visualization of crystal, volumetric and morphology data. J. Appl. Crystallogr. **44**(6), 1272–1276 (2011). 10.1107/s0021889811038970

[34] T. Li, X. Xia, J. Liu, Z. Liu, S. Hu et al., Suppressing surface lattice oxygen evolution by fluorinated graphene-scaffolded lithium-rich manganese-based cathode for enhanced stability. Energy Storage Mater. **49**, 555–563 (2022). 10.1016/j.ensm.2022.05.002

[35] C. Yin, Z. Wei, M. Zhang, B. Qiu, Y. Zhou et al., Structural insights into composition design of Li-rich layered cathode materials for high-energy rechargeable battery. Mater. Today **51**, 15–26 (2021). 10.1016/j.mattod.2021.10.020

[36] Z. Ye, B. Zhang, T. Chen, Z. Wu, D. Wang et al., A simple gas–solid treatment for surface modification of Li-rich oxides cathodes. Angew. Chem. Int. Ed. **60**(43), 23248–23255 (2021). 10.1002/anie.202107955 10.1002/anie.202107955 34405936

[37] H. Wu, J. Dong, Y. Zhang, L. Lin, G. Gao et al., Lattice oxygen redox reversibility modulation in enhancing the cycling stability of Li-rich cathode materials. Adv. Funct. Mater. **33**(41), 2303707 (2023). 10.1002/adfm.202303707

[38] Y. Wei, J. Cheng, D. Li, Y. Li, Z. Zeng et al., A structure self-healing Li-rich cathode achieved by lithium supplement of Li-rich LLZO coating. Adv. Funct. Mater. **33**(22), 2214775 (2023). 10.1002/adfm.202214775

[39] J. Zhang, F. Cheng, S. Chou, J. Wang, L. Gu et al., Tuning oxygen redox chemistry in Li-rich Mn-based layered oxide cathodes by modulating cation arrangement. Adv. Mater. **31**(42), 1901808 (2019). 10.1002/adma.201901808 10.1002/adma.201901808 31475397

[40] J. Song, H. Wang, Y. Zuo, K. Zhang, T. Yang et al., Building better full manganese-based cathode materials for next-generation lithium-ion batteries. Electrochem. Energy Rev. **6**(1), 20 (2023). 10.1007/s41918-023-00184-8

[41] X. Yang, K. Doyle-Davis, X. Gao, X. Sun, Recent progress and perspectives on designing high-performance thick electrodes for all-solid-state lithium batteries. eTransportation **11**, 100152 (2022). 10.1016/j.etran.2021.100152

[42] Y. Kuang, C. Chen, D. Kirsch, L. Hu, Thick electrode batteries: principles, opportunities, and challenges. Adv. Energy Mater. **9**(33), 1901457 (2019). 10.1002/aenm.201901457

[43] I. Kochetkov, T.-T. Zuo, R. Ruess, B. Singh, L. Zhou et al., Different interfacial reactivity of lithium metal chloride electrolytes with high voltage cathodes determines solid-state battery performance. Energy Environ. Sci. **15**(9), 3933–3944 (2022). 10.1039/d2ee00803c

[44] X. Wang, Q. Zhang, C. Zhao, H. Li, B. Zhang et al., Achieving a high-performance sodium-ion pouch cell by regulating intergrowth structures in a layered oxide cathode with anionic redox. Nat. Energy **9**(2), 184–196 (2024). 10.1038/s41560-023-01425-2

[45] S. Sun, C.-Z. Zhao, G.-Y. Liu, S.-C. Wang, Z.-H. Fu et al., Boosting anionic redox reactions of Li-rich cathodes through lattice oxygen and Li-ion kinetics modulation in working all-solid-state batteries. Adv. Mater. **37**(6), 2414195 (2025). 10.1002/adma.202414195 10.1002/adma.202414195 39696937

[46] W.-Z. Liu, X.-H. Meng, Z.-Y. Zhou, Q. Zheng, J.-L. Shi et al., Alleviating the sluggish kinetics of all-solid-state batteries *via* cathode single-crystallization and multi-functional interface modification. J. Energy Chem. **98**, 123–133 (2024). 10.1016/j.jechem.2024.06.014

[47] Y. Lu, C.-Z. Zhao, R. Zhang, H. Yuan, L.-P. Hou et al., The carrier transition from Li atoms to Li vacancies in solid-state lithium alloy anodes. Sci. Adv. **7**(38), eabi5520 (2021). 10.1126/sciadv.abi5520 34524850 10.1126/sciadv.abi5520 PMC8443184

[48] Y. Lu, C.-Z. Zhao, J.-Q. Huang, Q. Zhang, The timescale identification decoupling complicated kinetic processes in lithium batteries. Joule **6**(6), 1172–1198 (2022). 10.1016/j.joule.2022.05.005

[49] Y. Yang, C. Gao, T. Luo, J. Song, T. Yang et al., Unlocking the potential of Li-rich Mn-based oxides for high-rate rechargeable lithium-ion batteries. Adv. Mater. **35**(52), e2307138 (2023). 10.1002/adma.202307138 37689984 10.1002/adma.202307138

[50] Y. Pei, Q. Chen, M. Wang, B. Li, P. Wang et al., Reviving reversible anion redox in 3d-transition-metal Li rich oxides by introducing surface defects. Nano Energy **71**, 104644 (2020). 10.1016/j.nanoen.2020.104644

[51] J. Guo, Y. Lai, X. Gao, S. Li, H. Zhang et al., Triggering cationic/anionic hybrid redox stabilizes high-temperature Li-rich cathodes materials *via* three-in-one strategy. Energy Storage Mater. **69**, 103383 (2024). 10.1016/j.ensm.2024.103383

[52] J. Zhang, Q. Zhang, D. Wong, N. Zhang, G. Ren et al., Addressing voltage decay in Li-rich cathodes by broadening the gap between metallic and anionic bands. Nat. Commun. **12**(1), 3071 (2021). 10.1038/s41467-021-23365-9 34031408 10.1038/s41467-021-23365-9 PMC8144552

[53] A. Zimina, A. Léon, R. Steininger, Chemical bonding effects in Sc compounds studied using X-ray absorption and X-ray photoelectron spectroscopies. Phys. Chem. Chem. Phys. **26**(3), 2613–2621 (2024). 10.1039/d3cp04108e 38173391 10.1039/d3cp04108e

[54] X. Ding, D. Luo, J. Cui, H. Xie, Q. Ren et al., An ultra-long-life lithium-rich Li_1.2_Mn_0.6_Ni_0.2_O_2_ cathode by three-in-one surface modification for lithium-ion batteries. Angew. Chem. Int. Ed. **59**(20), 7778–7782 (2020). 10.1002/anie.202000628 10.1002/anie.202000628 32030866

[55] B. Li, H. Yan, J. Ma, P. Yu, D. Xia et al., Manipulating the electronic structure of Li-rich manganese-based oxide using polyanions: towards better electrochemical performance. Adv. Funct. Mater. **24**(32), 5112–5118 (2014). 10.1002/adfm.201400436

[56] L. Wang, L. Zhang, J. Li, J. Gao, C. Jiang et al., First-principles study of doping in LiMnPO_4_. Int. J. Electrochem. Sci. **7**(4), 3362–3370 (2012). 10.1016/S1452-3981(23)13961-7

