# Supplementary material for: Modulating Lattice Oxygen and Transport Kinetics of Li-Rich Cathodes in All-Solid-State Batteries Through Multifunctional Li3ScF6 Protective Layer
Source: Nanomicro Lett. 2026 May 21;18:383. doi: 10.1007/s40820-026-02209-5 (PMC13194812; doi:10.1007/s40820-026-02209-5)
Supplement: Supplementary file 1 — Supplementary file1 (DOCX 17310 kb) [file 40820_2026_2209_MOESM1_ESM.docx]

Supporting Information for

**Modulating Lattice Oxygen and Transport Kinetics of Li-Rich Cathodes in All-Solid-State Batteries through Multifunctional Li_3_ScF_6_ Protective Layer**

Peng Lei^1^, Gang Wu^1^, Xiang Qi^1^, Yang Li^1^, Meng Wu^1^, Wanqing Ren^1^, Huan Li^1^, Lei Gao^1^, Dan Zhou^1^*, Li-Zhen Fan^1^*

^1^Institute of Advanced Materials and Technology, University of Science and Technology Beijing, Beijing 100083, P. R. China

*Corresponding authors. E-mail: fanlizhen@ustb.edu.cn (Li-Zhen Fan); zhoudan@ustb.edu.cn (Dan Zhou)

**Supplementary Figures and Tables**

**
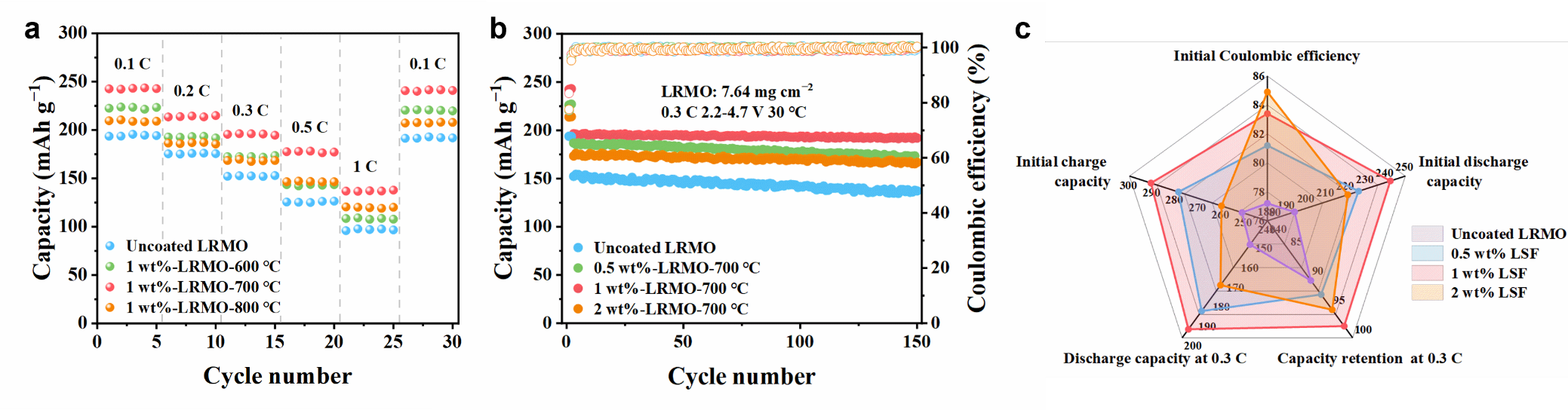
**

**Fig. S1** **a**) Rate performance comparison of ASSLBs with different thermal temperature-treated LRMO. **b**) Cycling performance comparison of ASSLBs with different LSF coating amounts. **c**) Radar chart for comprehensive performance comparison of ASSLBs with different LSF coating amounts

**

**

**Fig. S2** Raman spectra of B-LRMO and C-LRMO


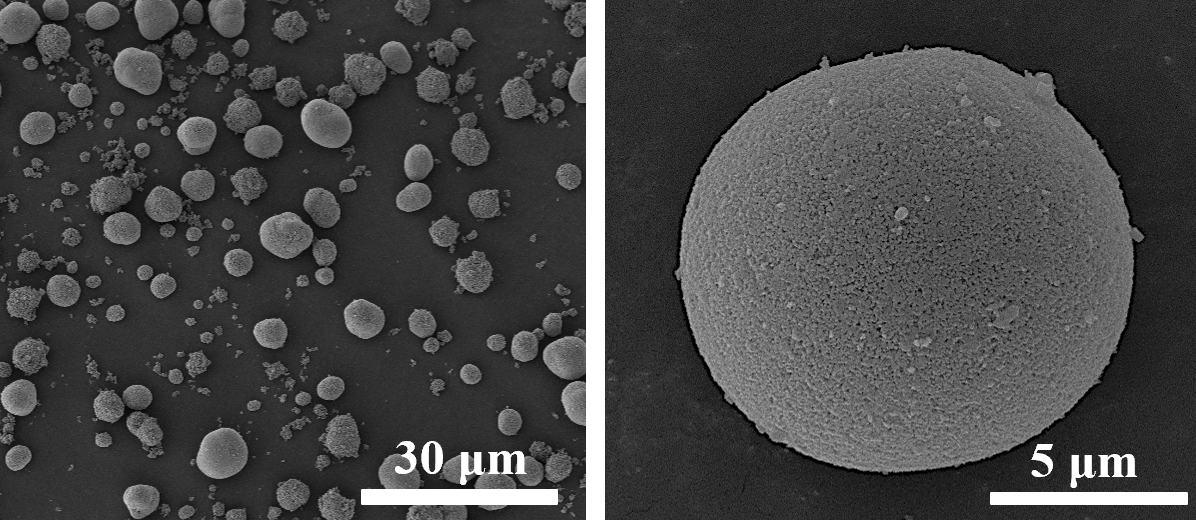


**Fig. S3** SEM images of B-LRMO particles


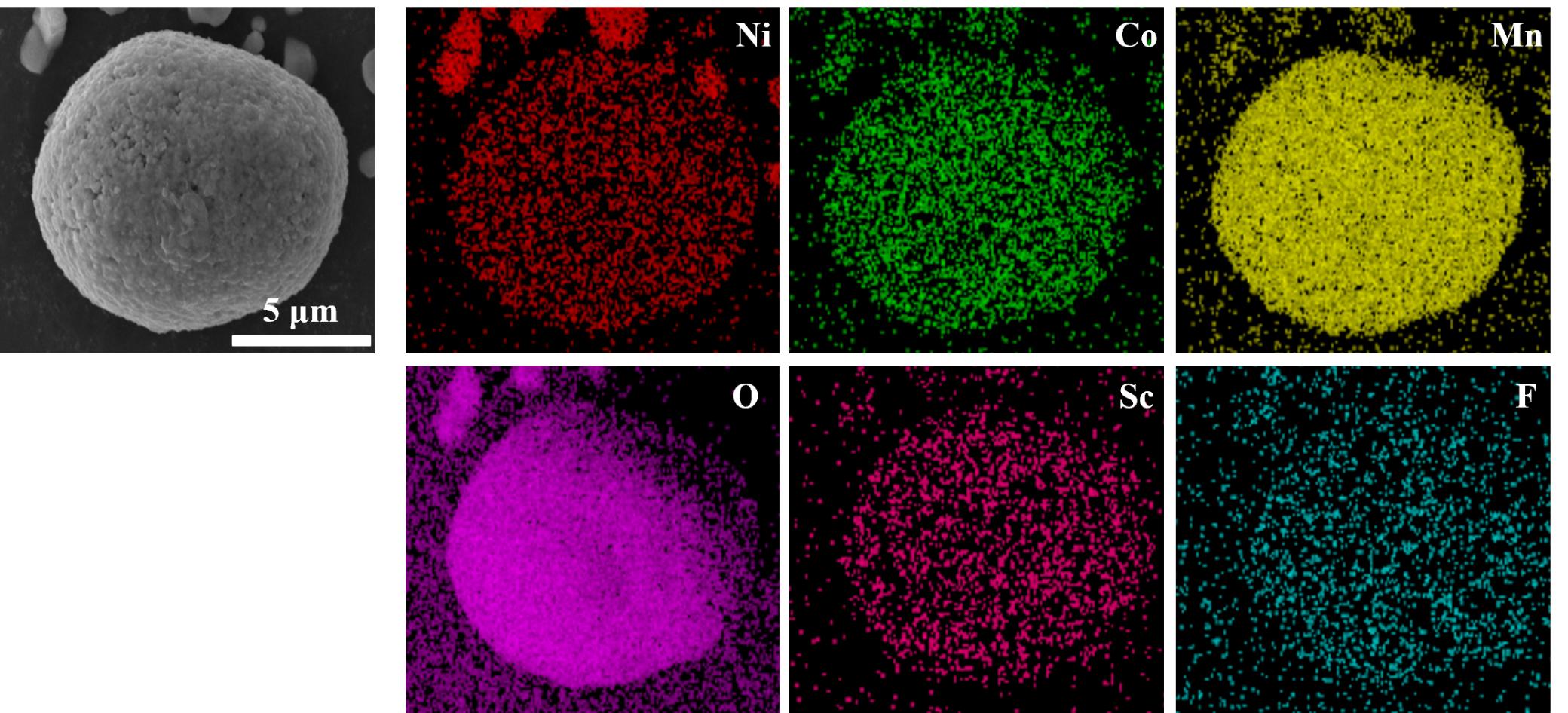


**Fig. S4** SEM image of C-LRMO particles and corresponding EDS mappings of Ni, Co, Mn, O, Sc, and F elements


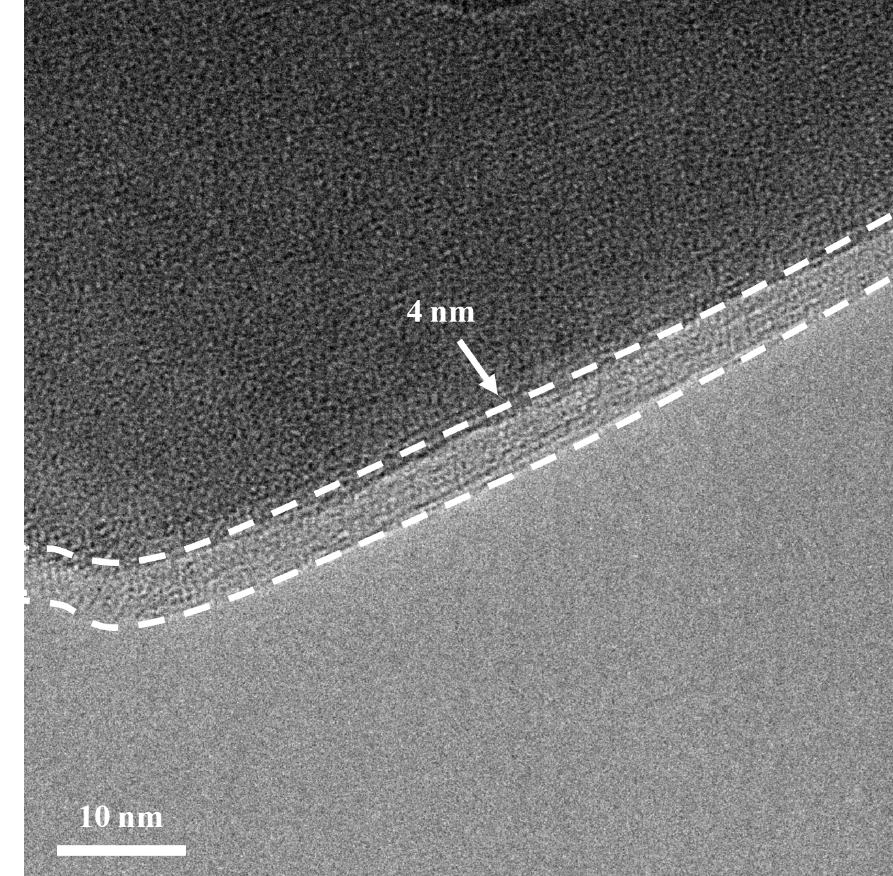


**Fig. S5** HRTEM image of C-LRMO powder


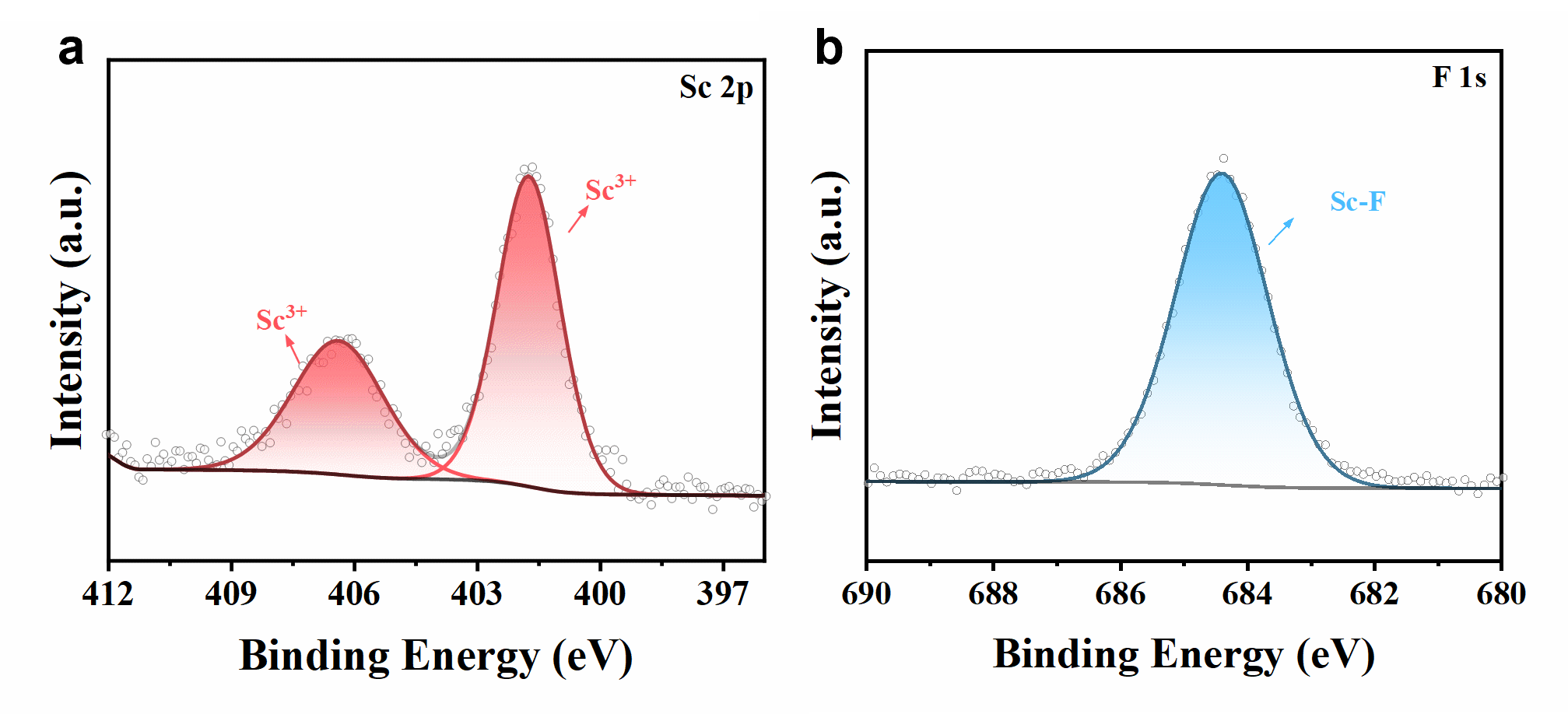


**Fig. S6** XPS spectra of **a**) Sc 2p and **b**) F 1s for C-LRMO


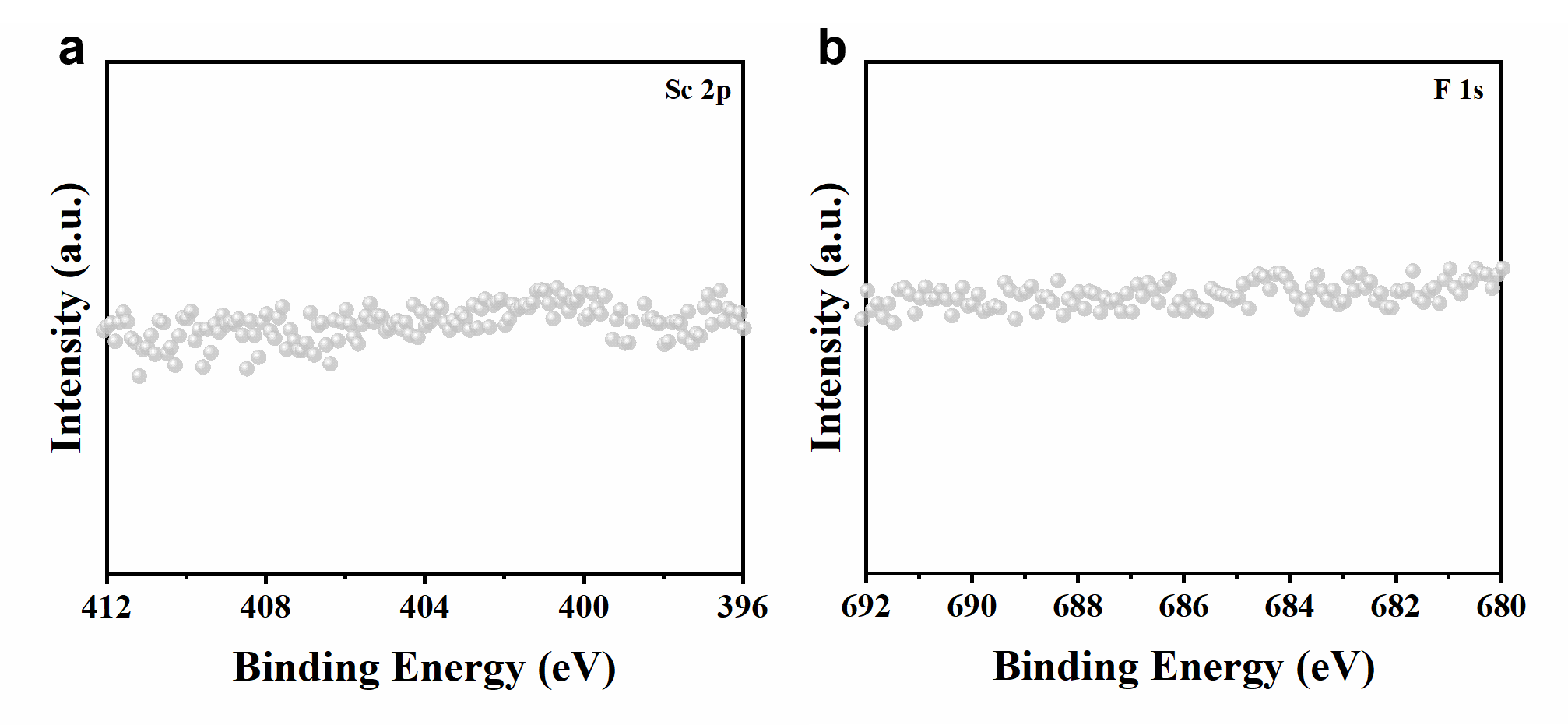


**Fig. S7** XPS spectra of **a**) Sc 2p and **b**) F 1s for B-LRMO

**
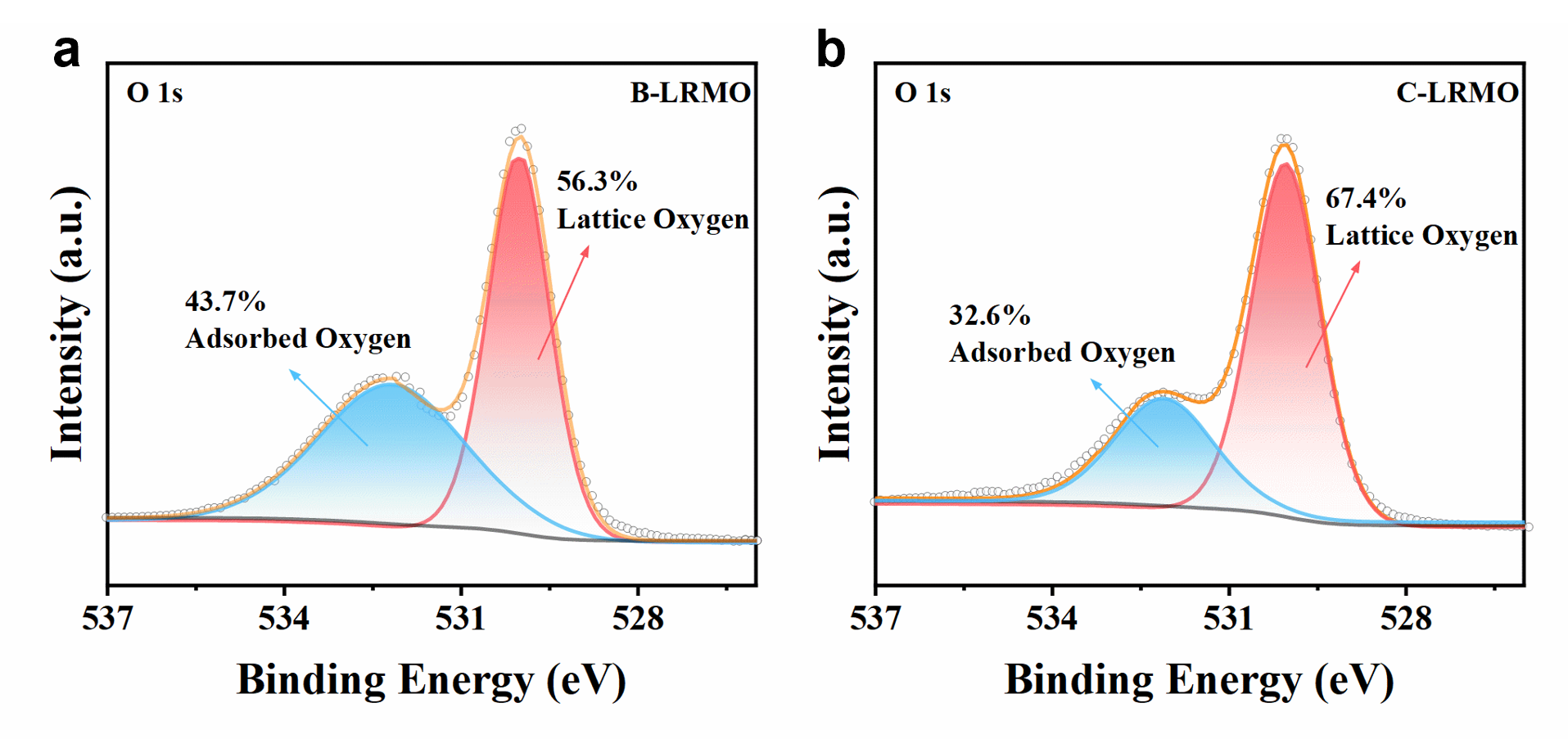
**

**Fig. S8** O 1s XPS spectra of **a**) B-LRMO and **b**) C-LRMO

**
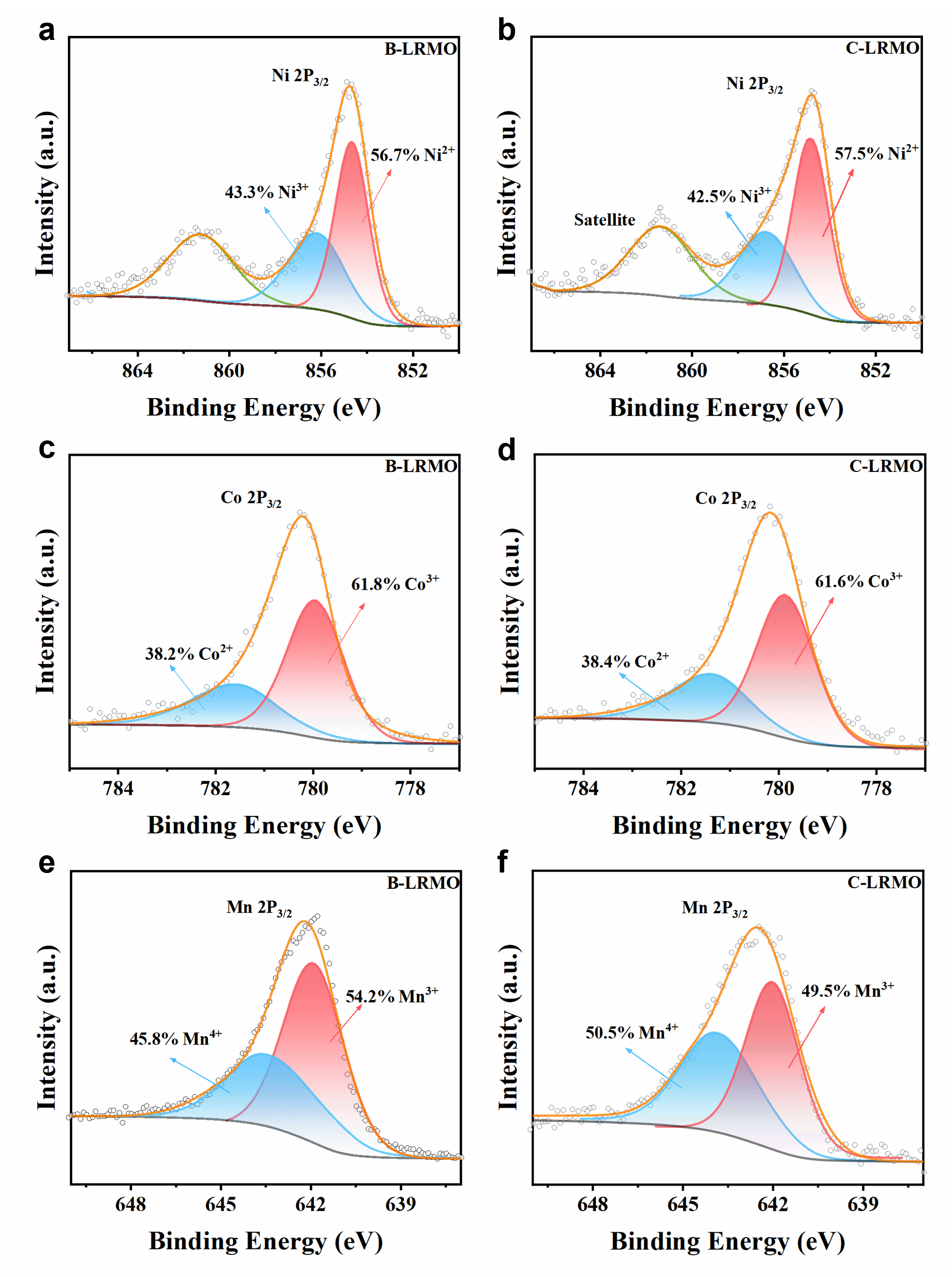
**

**Fig. S9** The Ni 2p XPS spectra of **a**) B-LRMO and **b**) C-LRMO, Co 2p XPS spectra of **c**) B-LRMO and **d**) C-LRMO, Mn 2p XPS spectra of **e**) B-LRMO and f) C-LRMO


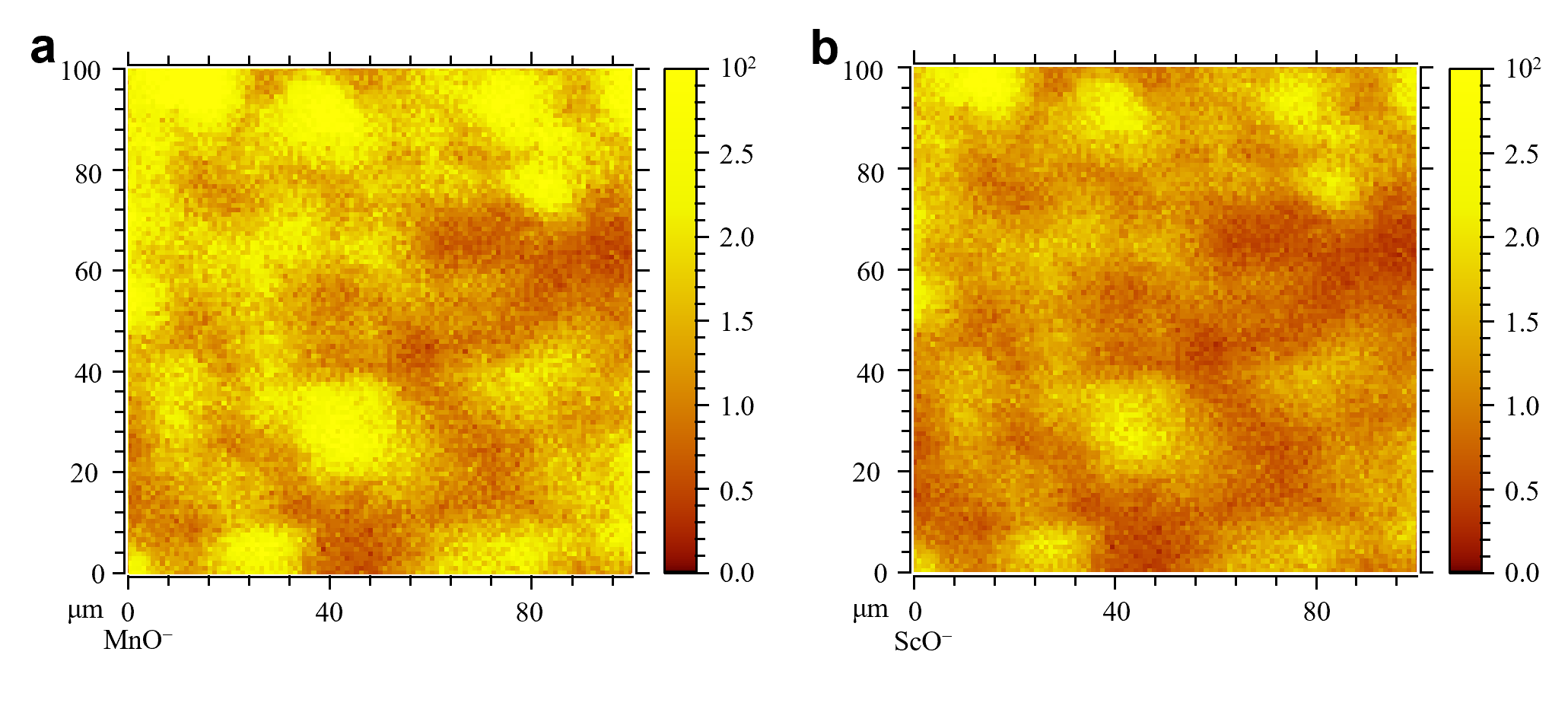
 **Fig. S10** TOF-SIMS images of **a**) MnO^−^ and **b**) ScO^−^ species in the C-LRMO cathode


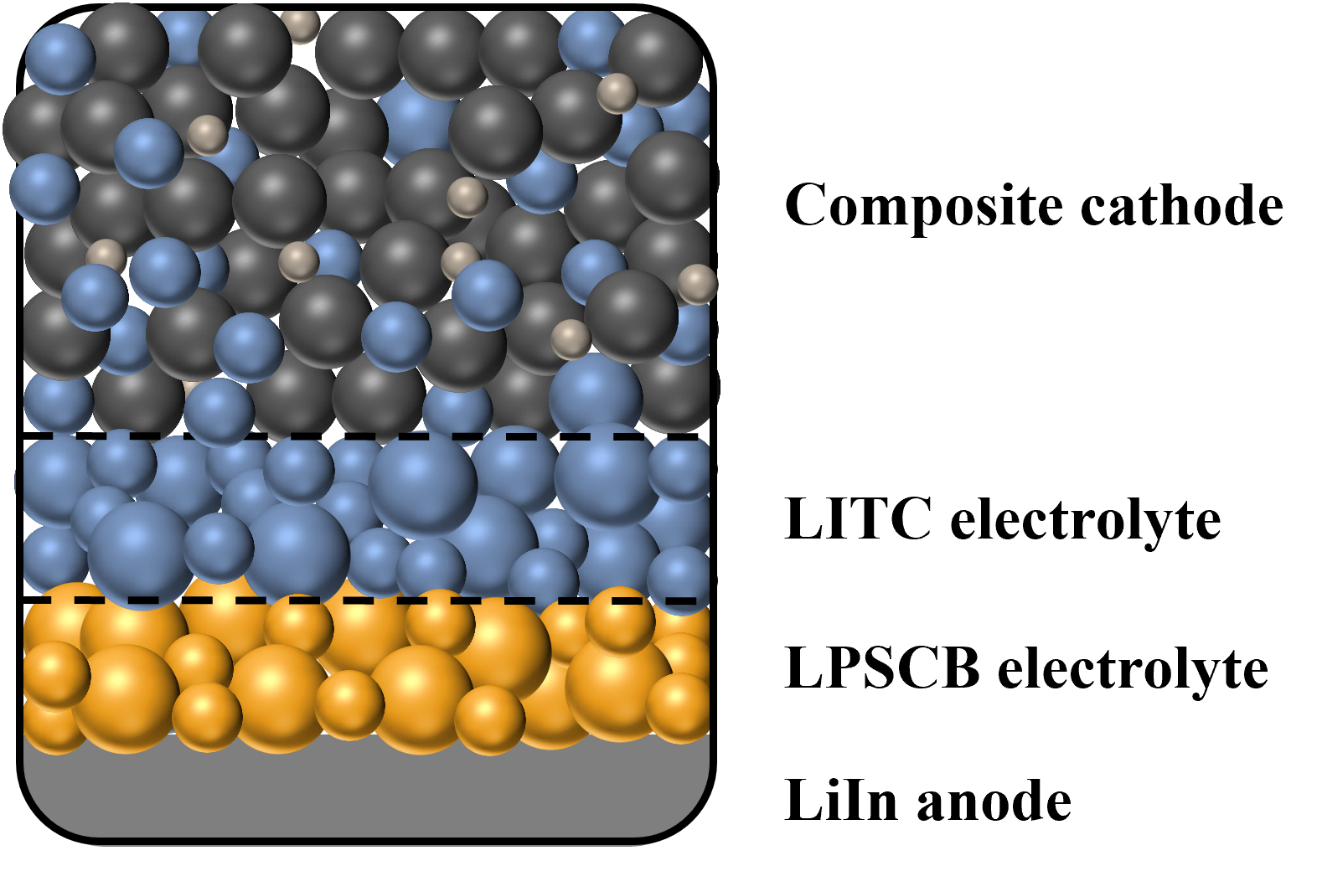


**Fig. S11** The schematic illustration of the ASSLBs


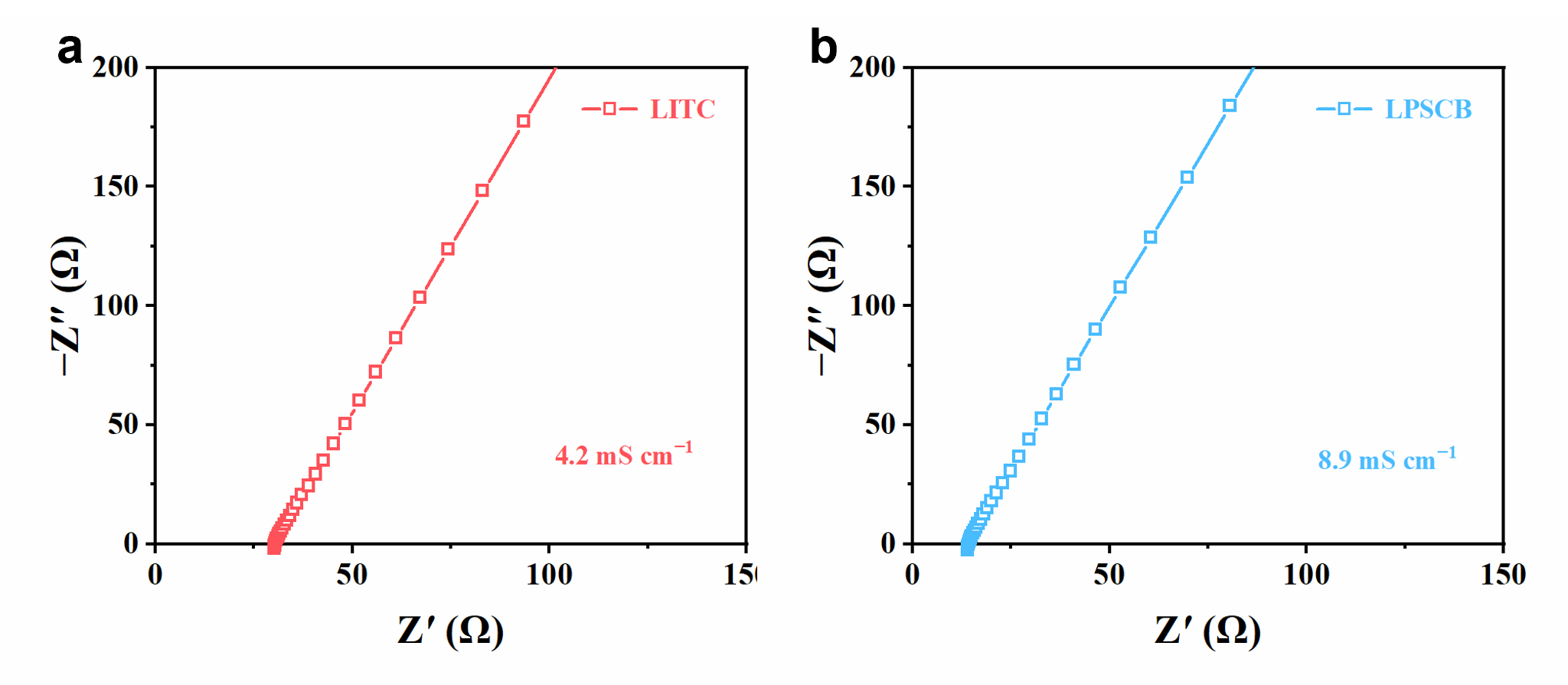


**Fig. S12** Nyquist plots of **a**) LITC and **b**) LPSCB at 30 ℃


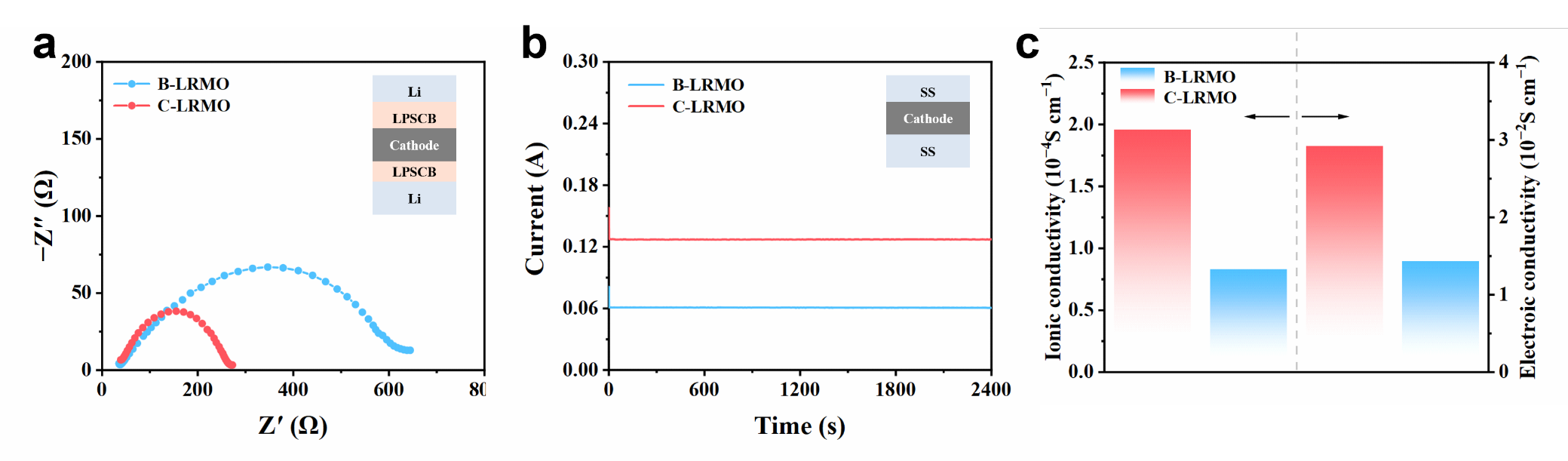


**Fig. S13** **a**) Nyquist plots and **b**) DC polarization curves of B-LRMO and C-LRMO composite cathode, and **c**) corresponding ionic or electronic conductivity


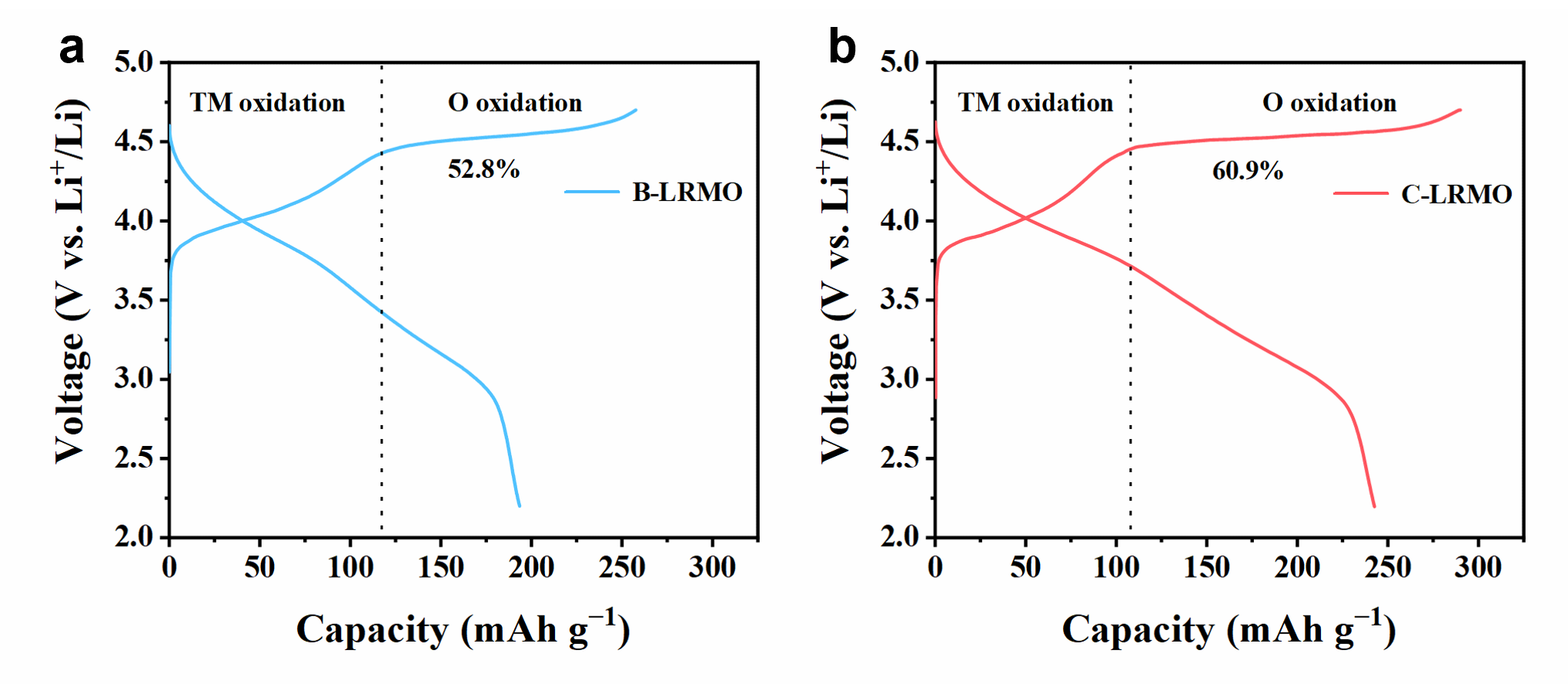


**Fig. S14** First-cycle charge/discharge voltage profiles of **a**) B-LRMO and **b**) C-LRMO at 0.1 C. A voltage of ~4.47 V was selected to distinguish TM oxidation and O oxidation





**Fig. S15** CV curves of B-LRMO and C-LRMO





**Fig. S16** The cycling voltage decay of B-LRMO and C-LRMO at 0.3 C





**Fig. S17** Long-term cycling stability of C-LRMO at 1 C with cathode mass loading of 7.64 mg cm^−2^


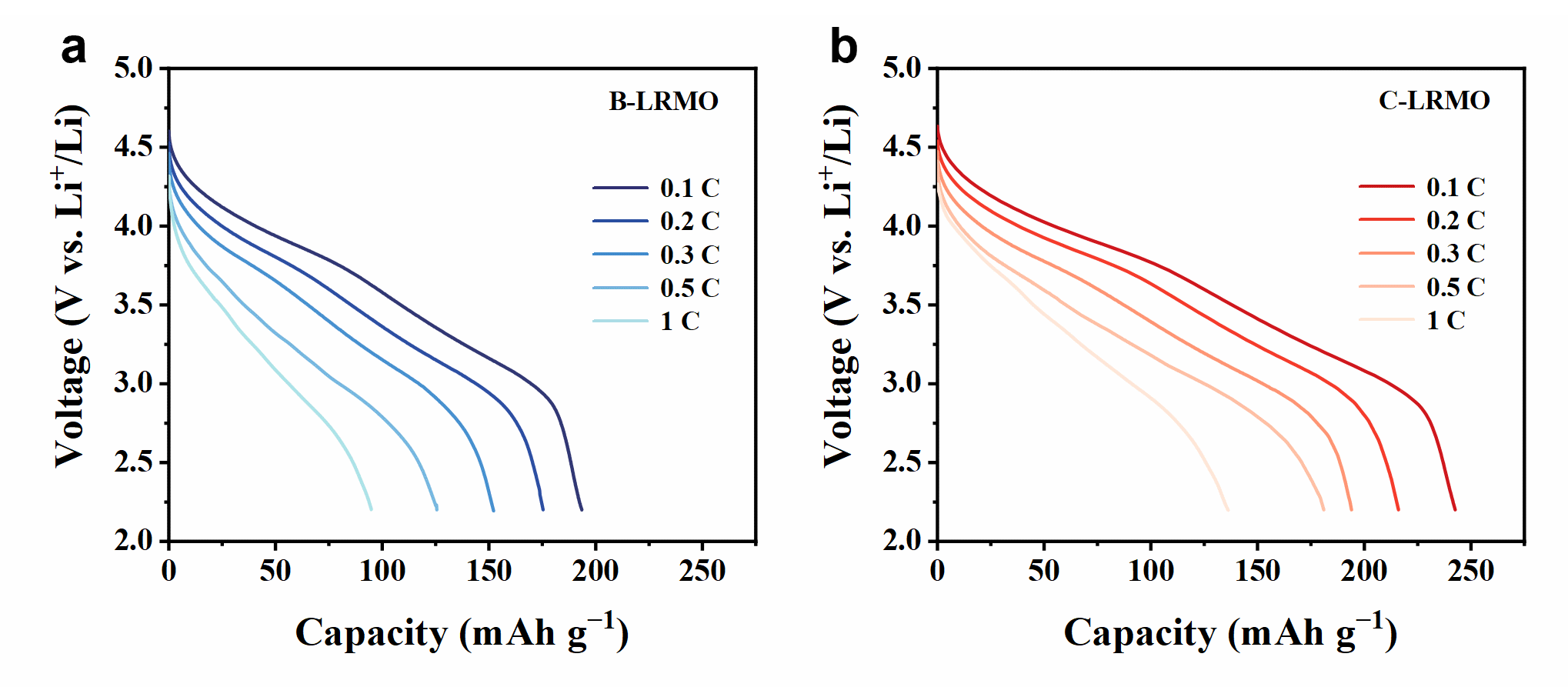


**Fig. S18** Discharge voltage curves of **a**) B-LRMO and **b**) C-LRMO electrodes at different current densities


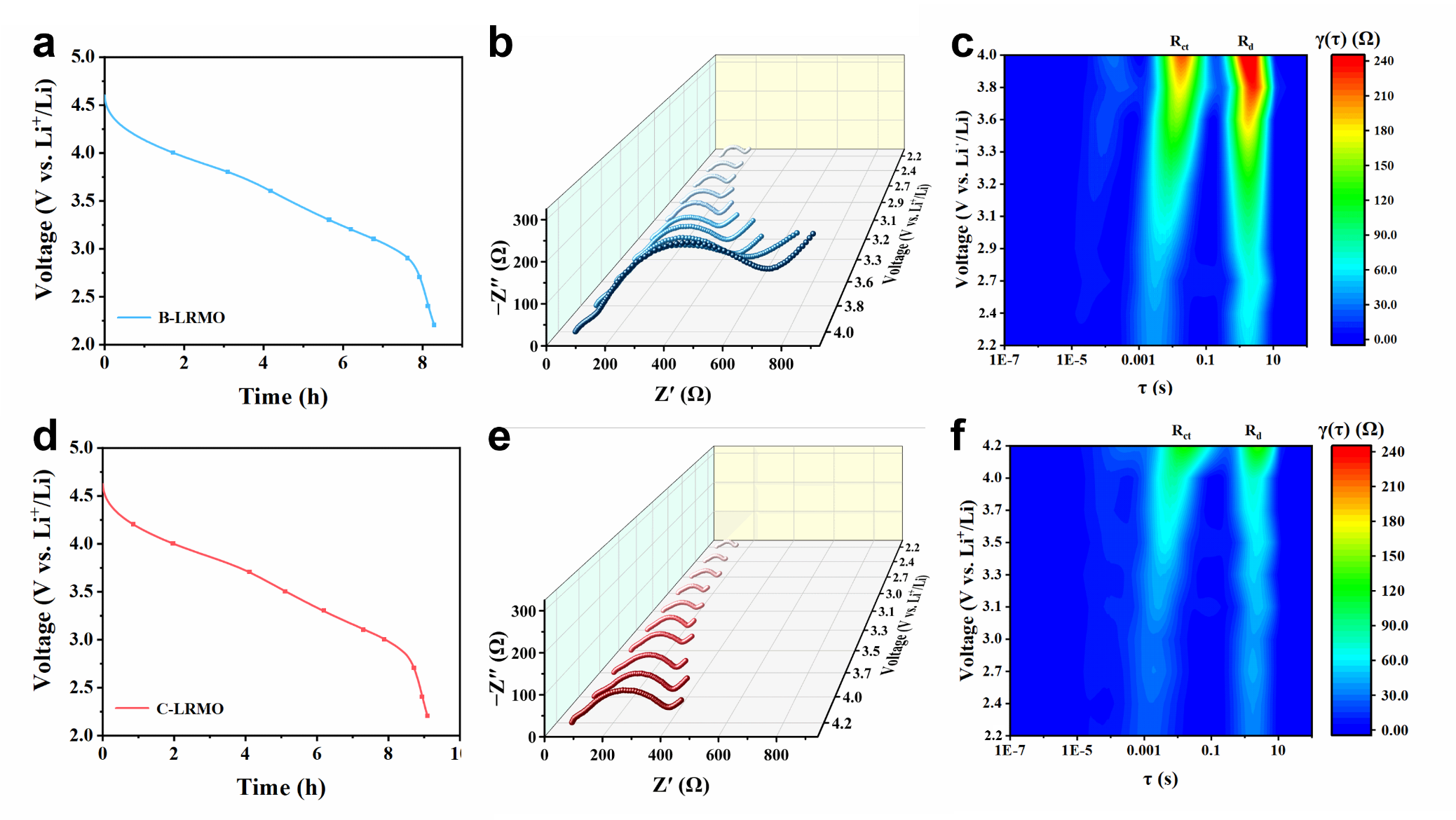


**Fig. S19** **a**) Initial discharging voltage profile of the B-LRMO ASSLBs between 2.2 and 4.7 V versus Li^+^/Li. **b**) Interfacial impedance evolution of the B-LRMO ASSLBs and **c**) corresponding DRT profile transformation derived from in situ EIS. **d**) Initial discharging voltage profile of the C-LRMO ASSLBs between 2.2 and 4.7 V versus Li⁺/Li. **e**) Interfacial impedance evolution of the C-LRMO ASSLBs and **f**) corresponding DRT profile transformation derived from in situ EIS

**
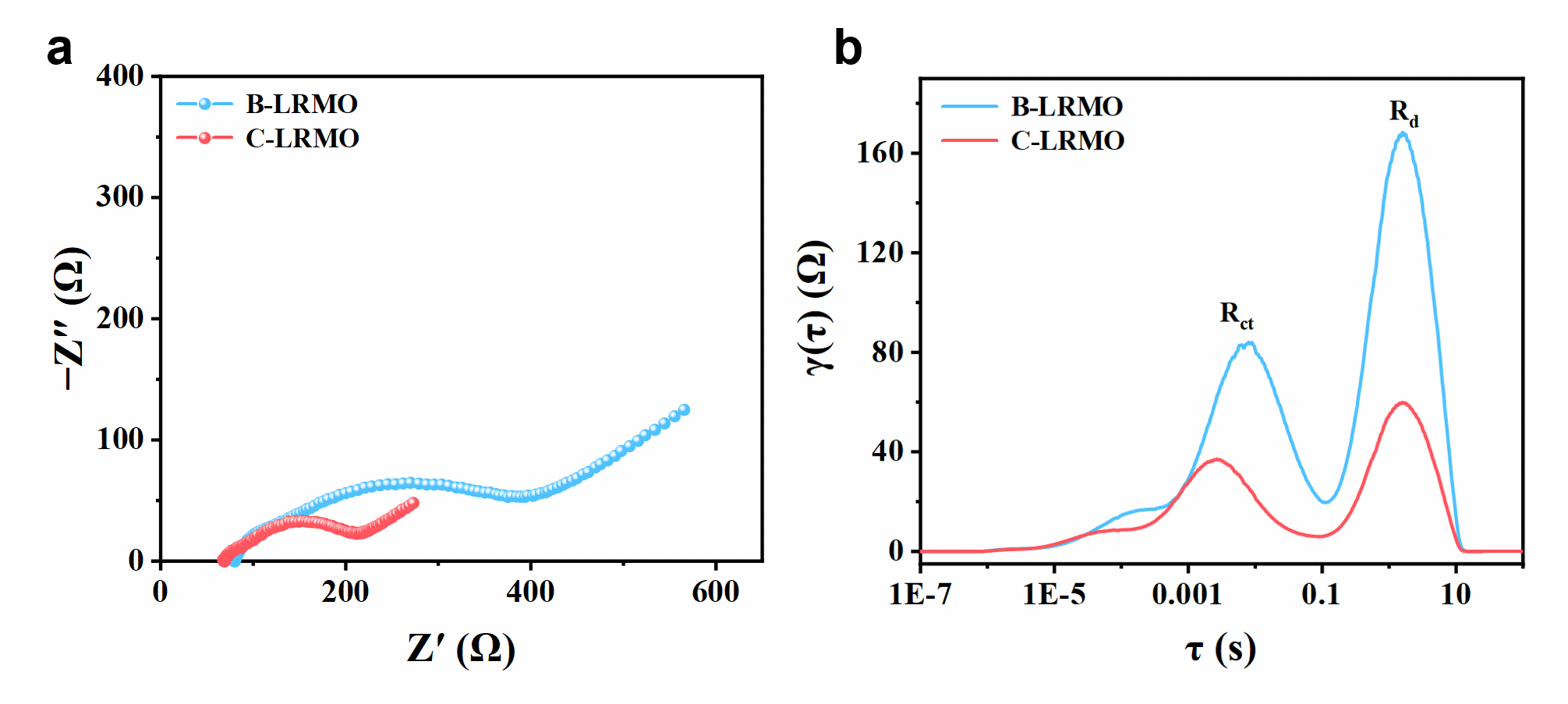
**

**Fig. S20** **a**) Nyquist plots of B-LRMO and C-LRMO cells after 500 cycles at 0.3 C and **b**) the comparison of DRT profiles transformation of EIS


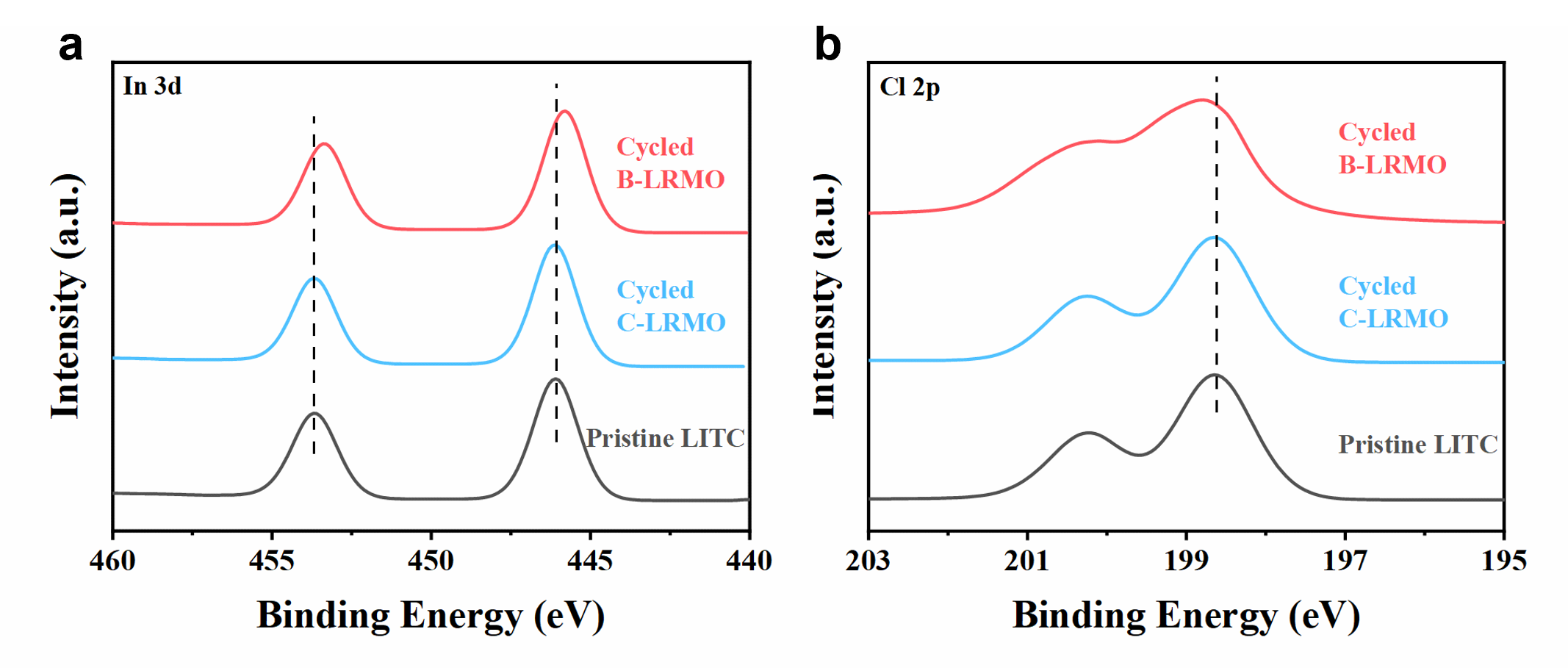


**Fig. S21** XPS spectra of **a**) In 3d and **b**) Cl 2p in the composite cathodes before and after 500 cycles at 0.3 C


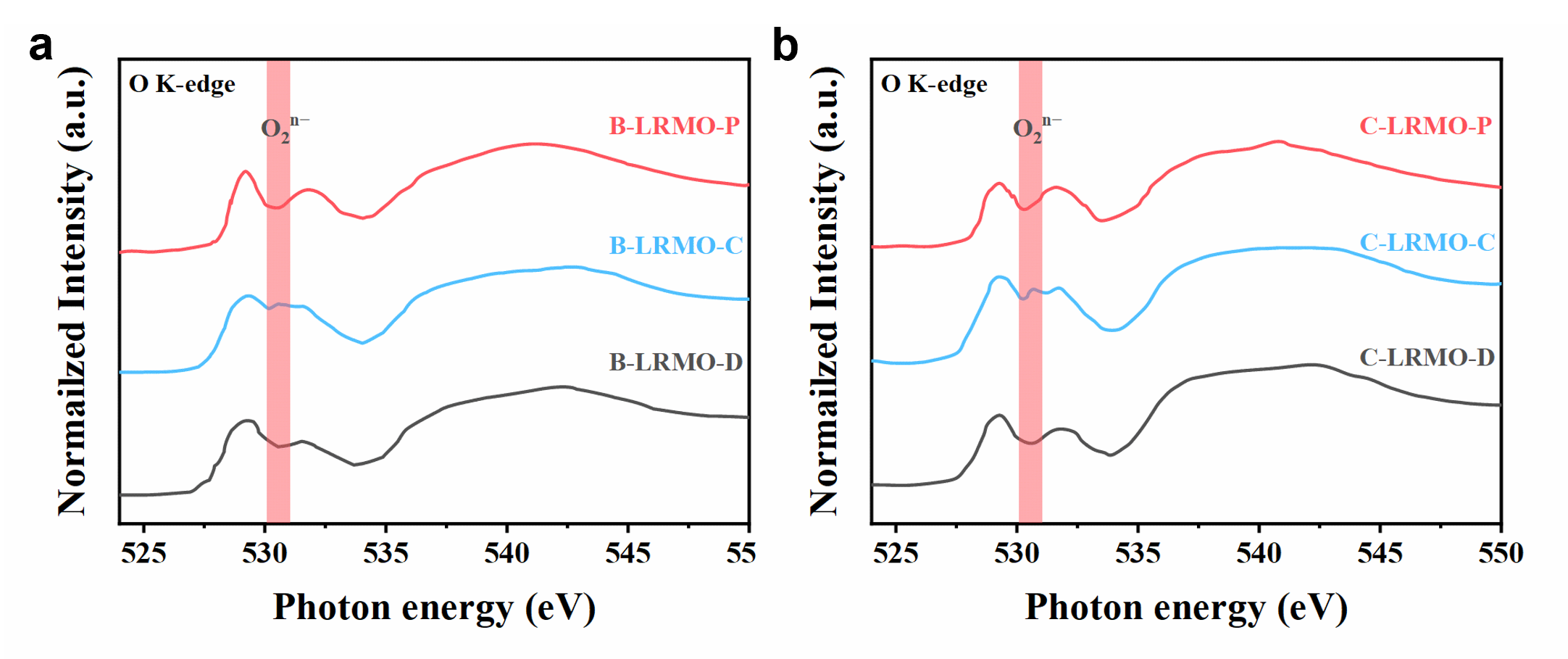


**Fig. S22** O K-edge XANES spectra of **a**) B-LRMO and **b**) C-LRMO at different voltage states


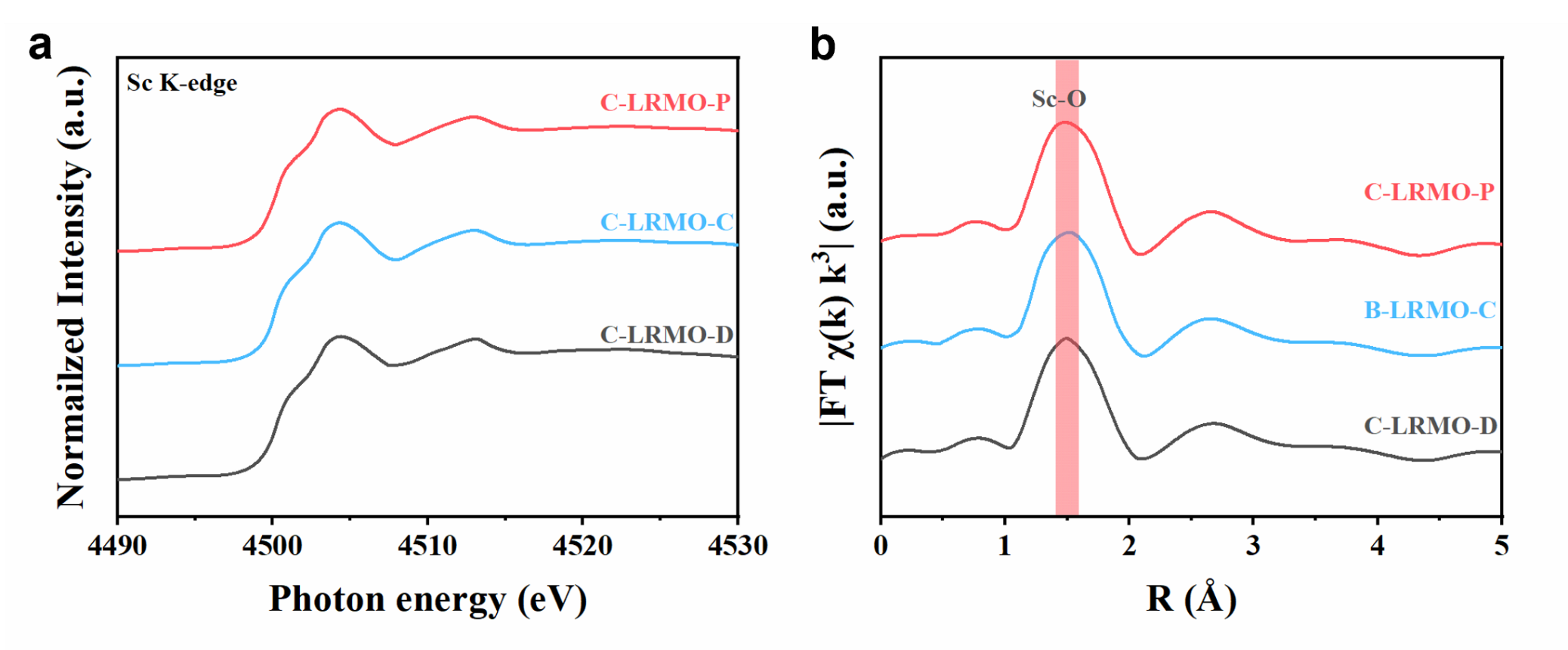


**Fig. S23** Sc K-edge spectra of C-LRMO at different voltage states: **a**) XANES spectra and **b**) EXAFS spectra


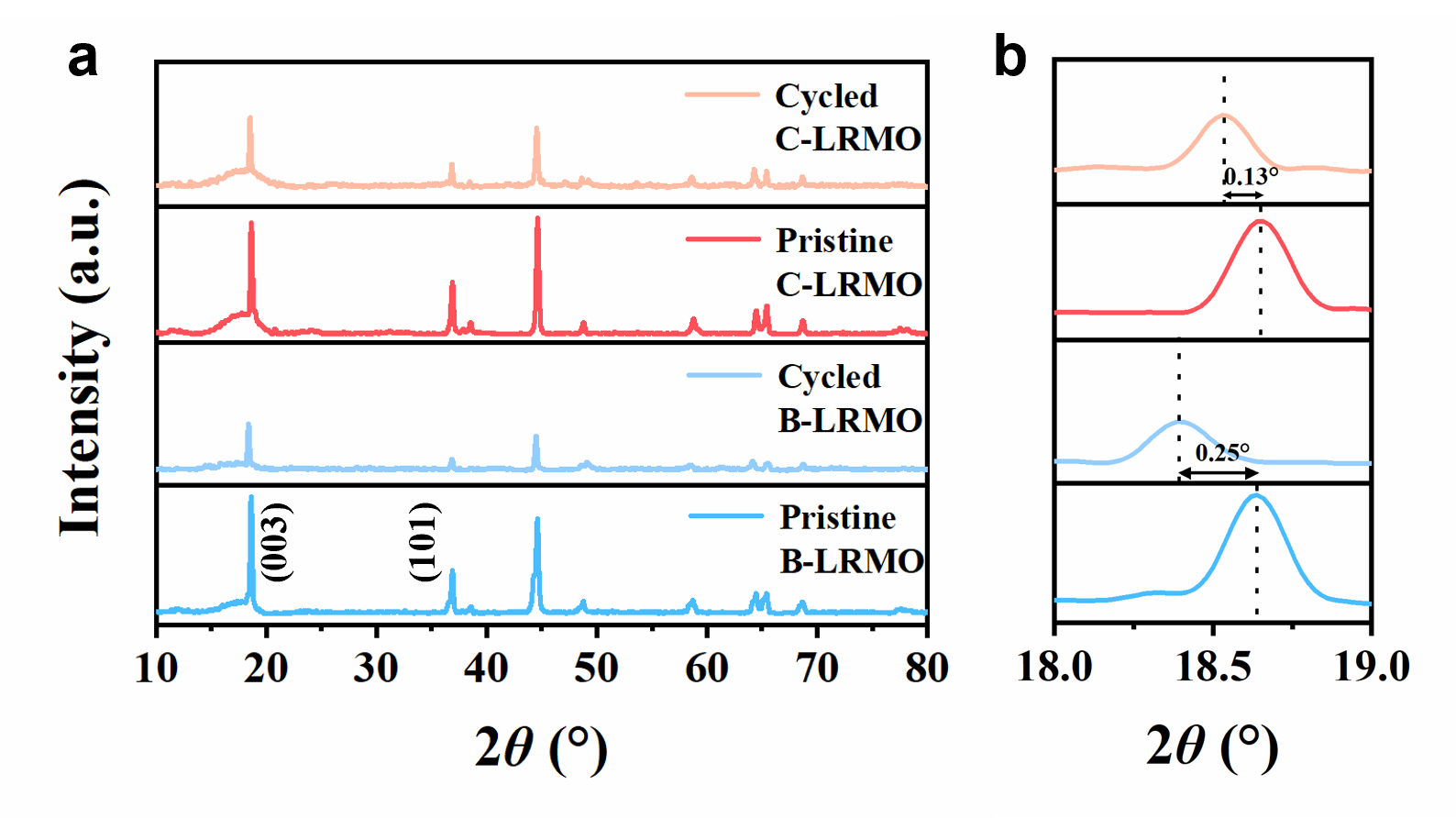


**Fig. S24** The XRD patterns of **a**) the overall and **b**) the enlarged patterns of the (003) peak for the cycled and pristine cathodes of B-LRMO and C-LRMO


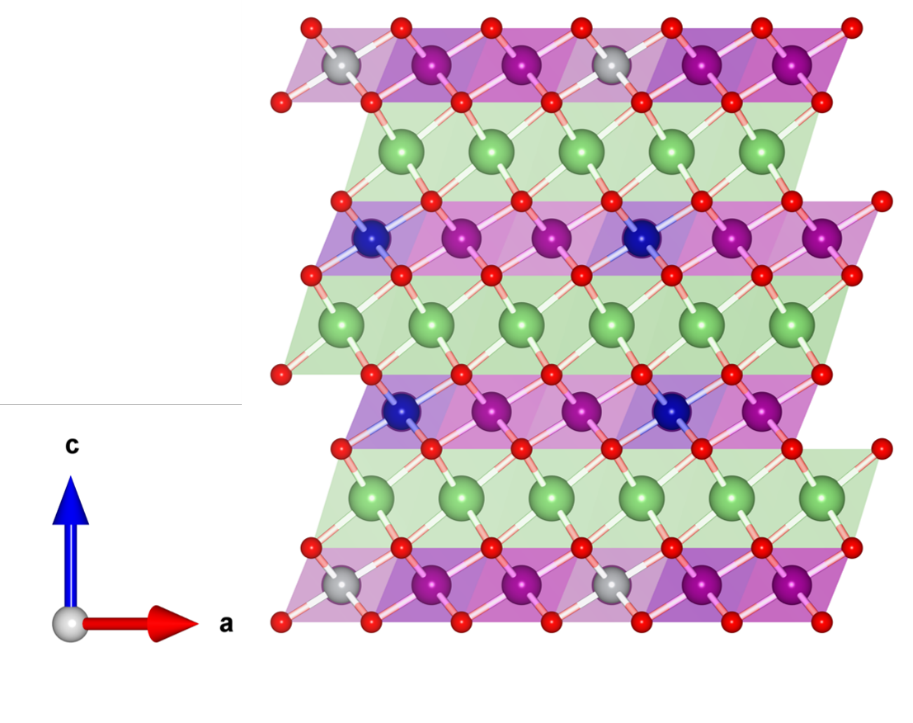


**Fig. S25** Relaxed crystal structures of B-LRMO. The color code for atoms: red, O; green, Li; gray, Ni; blue, Co; purple, Mn


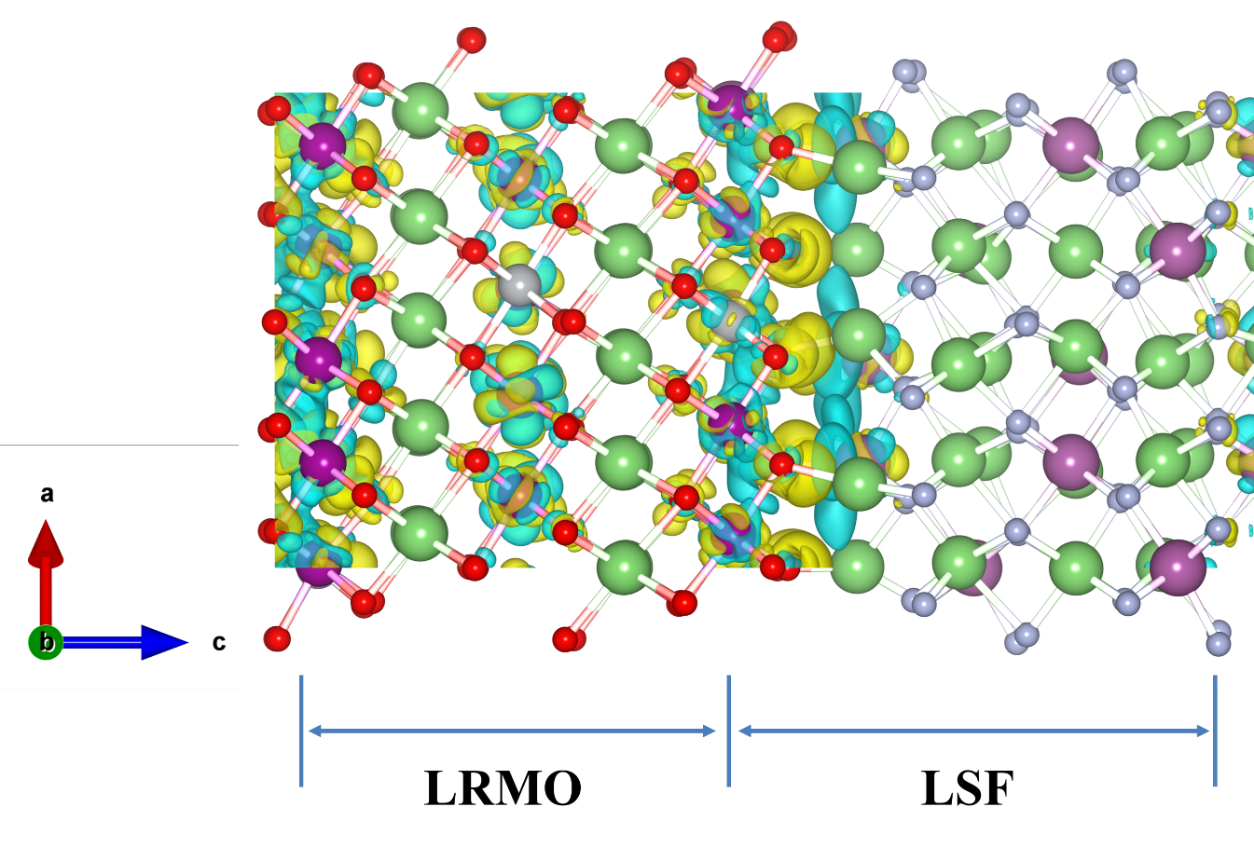


**Fig. S26** Charge density difference of the C-LRMO. The color code for atoms: red, O; green, Li; gray, Ni; blue, Co; purple, Mn; orchid purple, Sc; periwinkle blue, F





**Fig. S27** Calculated mutual reaction energies of different interfaces





**Fig. S28** The calculated thermodynamic electrochemical stability windows of LITC and LSF

**Table S1** Lattice parameters of B-LRMO and C-LRMO derived from Rietveld refinement of XRD patterns

| Sample | Space  group | Lattice parameters | | | | | | Vol  (Å^3^) |
| --- | --- | --- | --- | --- | --- | --- | --- | --- |
|  |  | a(Å) | b(Å) | c(Å) | α(˚) | β(˚) | γ(˚) |  |
| B-LRMO | R$\bar{3}$m | 2.85443 | 2.85443 | 14.26466 | 90.0000 | 90.0000 | 120.0000 | 100.654 |
| B-LRMO | C2/m | 4.92549 | 8.55097 | 5.02809 | 90.0000 | 109.2358 | 90.0000 | 199.948 |
| C-LRMO | R$\bar{3}$m | 2.85166 | 2.85166 | 14.21446 | 90.0000 | 90.0000 | 120.0000 | 100.105 |
| C-LRMO | C2/m | 4.92893 | 8.55104 | 5.02811 | 90.0000 | 109.2472 | 90.0000 | 200.077 |

**Table S2** Comparison of the electrochemical performance for the LRMO-based ASSLBs

| Cathode materials | Solide  electrolyte in  cathode | Areal  Capacity  (mAh cm^−2^) | Cycle  number | Capacity retention (%) | Temperature  (℃) | Ref. |
| --- | --- | --- | --- | --- | --- | --- |
| LLO-Ru@S | Li_6_PS_5_Cl | 0.65 | 1.0 C  2022 cycles | 72.0 | 55 | [S1] |
| SC-LRMO | Li_6_PS_5_Cl | 1.2 | 1.0 C  300 cycles | 86.0 | 60 | [S2] |
| LRCo10@5LN | BM-Li_6_PS_5_Cl | 1.9 | 0.5 C  300 cycles | 85.0 | 25 | [S3] |
| Li_1.14_Ni_0.29_Mn_0.57_O_2_ | LiAlOCl | 2.0 | 0.3 C  190 cycles | 92.7 | 25 | [S4] |
| S-LRMO | Li_3_InCl_4.8_F_1.2_ | 2.9 | 0.2 C  60 cycles | 80.3 | 25 | [S5] |
| SCM LLO | Li_3_InCl_6_ | 2.9 | 0.5 C  431 cycles | 60.0 | 25 | [S6] |
| 5W&LRMO | Li_3_InCl_6_ | 3.15 | 0.2 C  100 cycles | 84.1 | 25 | [S7] |
| LBO-LRMO | Li_3_InCl_6_ | 0.97 | 1.0 C  2000 cycles | 80.4 | 25 | [S8] |
| LMRO-0.6Li_2_MnO_3_/0.05LiNiO_2_ | Li_3_InCl_6_ | 1.96 | 0.2 C  1000 cycles | 87.0 | 25 | [S9] |
| **C-LRMO** | **Li_2.6_In_0.8_Ta_0.2_Cl_6_** | **1.85** | **1 C**  **1000 cycles** | **84.7** | **30** | **This work** |
|  |  | **4.17** | **0.1 C**  **300 cycles** | **81.1** | **60** |  |

**Supplementary References**

1. Y. Wang, D. Wu, P. Chen, P. Lu, X. Wang et al., Dual-function modifications for high-stability Li-rich cathode toward sulfide all-solid-state batteries. Adv. Funct. Mater. **34**(4), 2309822 (2024). <https://doi.org/10.1002/adfm.202309822>
2. Y. Wu, C. Li, X. Zheng, W. Zhao, H. Wang et al., High energy sulfide-based all-solid-state lithium batteries enabled by single-crystal Li-rich cathodes. ACS Energy Lett. **9**(10), 5156–5165 (2024). <https://doi.org/10.1021/acsenergylett.4c01764>
3. W. Du, Q. Shao, Y. Wei, C. Yan, P. Gao et al., High-energy and long-cycling all-solid-state lithium-ion batteries with Li- and Mn-rich layered oxide cathodes and sulfide electrolytes. ACS Energy Lett. **7**(9), 3006–3014 (2022). <https://doi.org/10.1021/acsenergylett.2c01637>
4. G. Wang, S. Zhang, H. Wu, M. Zheng, C. Zhao et al., Oxychloride polyanion clustered solid-state electrolytes *via* hydrate-assisted synthesis for all-solid-state batteries. Adv. Mater. **37**(4), 2410402 (2025). <https://doi.org/10.1002/adma.202410402>
5. S. Sun, C.-Z. Zhao, H. Yuan, Z.-H. Fu, X. Chen et al., Eliminating interfacial O-involving degradation in Li-rich Mn-based cathodes for all-solid-state lithium batteries. Sci. Adv. **8**(47), eadd5189 (2022). <https://doi.org/10.1126/sciadv.add5189>
6. R. Yu, C. Wang, H. Duan, M. Jiang, A. Zhang et al., Manipulating charge-transfer kinetics of lithium-rich layered oxide cathodes in halide all-solid-state batteries. Adv. Mater. **35**(5), e2207234 (2023). <https://doi.org/10.1002/adma.202207234>
7. W.-J. Kong, C.-Z. Zhao, L. Shen, S. Sun, X.-Y. Huang et al., Bulk/interfacial structure design of Li-rich Mn-based cathodes for all-solid-state lithium batteries. J. Am. Chem. Soc. **146**(41), 28190–28200 (2024). <https://doi.org/10.1021/jacs.4c08115>
8. S. Sun, C.-Z. Zhao, G.-Y. Liu, S.-C. Wang, Z.-H. Fu et al., Boosting anionic redox reactions of Li-rich cathodes through lattice oxygen and Li-ion kinetics modulation in working all-solid-state batteries. Adv. Mater. **37**(6), 2414195 (2025). <https://doi.org/10.1002/adma.202414195>
9. Z. Wu, Q. Shao, Y. Wei, C. Yan, P. Gao et al., Multi-strategies interface and structure design of Li- and Mn-rich layered oxide for all-solid-state lithium batteries. Nano Energy **122**, 109281 (2024). <https://doi.org/10.1016/j.nanoen.2024.109281>
